# Cigarette smoke extract impairs gingival epithelial barrier function

**DOI:** 10.1038/s41598-023-36366-z

**Published:** 2023-06-07

**Authors:** Shunsuke Yamaga, Keita Tanigaki, Eriko Nakamura, Naoko Sasaki, Yuta Kato, Masae Kuboniwa, Michiya Matsusaki, Atsuo Amano, Hiroki Takeuchi

**Affiliations:** 1 Department of Preventive Dentistry, Graduate School of Dentistry, Osaka University, Suita-Osaka, 565-0871 Japan; 2 Department of Preventive Dentistry, Osaka University Dental Hospital, 1–8 Yamadaoka, Suita-Osaka, 565-0871 Japan; 3 Joint Research Laboratory (TOPPAN) for Advanced Cell Regulatory Chemistry, Graduate School of Engineering, Osaka University, Suita-Osaka, 565-0871 Japan; 4 Department of Applied Chemistry, Graduate School of Engineering, Osaka University, Suita-Osaka, 565-0871 Japan

**Keywords:** Cell biology, Pathogenesis

## Abstract

We previously showed that junctional adhesion molecule 1 (JAM1) and coxsackievirus and adenovirus receptor (CXADR), tight junction-associated proteins, have important roles to maintain epithelial barrier function in gingival tissues. Smoking is considered to be a significant risk factor for periodontal disease. The present study was conducted to examine the effects of cigarette smoke extract (CSE) on JAM1 and CXADR in human gingival epithelial cells. CSE was found to cause translocation of JAM1 from the cellular surface to EGFR-positive endosomes, whereas CXADR did not. Using a three-dimensional multilayered gingival epithelial tissue model, CSE administration was found to increase permeability to lipopolysaccharide and peptidoglycan, whereas overexpression of JAM1 in the tissue model prevented penetration by those substrates. Furthermore, vitamin C increased JAM1 expression, and inhibited penetration of LPS and PGN induced by CSE. These findings strongly suggest that CSE disrupts gingival barrier function via dislocation of JAM1, thus allowing bacterial virulence factors to penetrate into subepithelial tissues. Furthermore, they indicate that vitamin C increases JAM1 expression and prevents disruption of gingival barrier function by CSE.

## Introduction

Periodontal disease is a chronic infectious disease caused by complex actions of periodontal bacteria in oral biofilm, with cigarette smoking recognized as significant among the many known risk factors^1–3^. Essentially, both natural tobacco leaves and smoke formed from burning tobacco contain several toxic chemicals. Studies of cigarette smoke extract (CSE) have shown that it causes increased permeability in respiratory epithelium and human bronchial epithelial cells, leading to impairment of epithelial barrier function^4,5^. As for the oral region, cigarette smoking reportedly inhibits gingival epithelial cell growth^6^. Furthermore, as compared to non-smokers, cigarette smokers are known to show worse response to periodontal treatment^7,8^. On the other hand, the molecular mechanisms associated with the negative influence of smoking on periodontal tissues are not well understood.

Human mucosal surfaces are exposed to abundant microbiota and their virulence factors. Lipopolysaccharides (LPS), endotoxins of gram-negative bacteria, and peptidoglycan (PGN) are prototypical representatives of pathogen-associated molecular patterns recognized by innate immunity factors^9^. Using a gingival epithelial tissue model, we previously showed that the periodontal pathogen *Porphyromonas gingivalis* specifically degraded two tight junction-associated proteins, junctional adhesion molecule 1 (JAM1) and coxsackievirus and adenovirus receptor (CXADR), which allowed the pathogen to successfully break down the gingival epithelial barrier, thus increasing epithelial permeability to LPS and PGN, which likely leads to onset of periodontal disease^10–12^. JAM1 and CXADR are thought to play important roles in maintenance of the epithelial barrier to prevent periodontal diseases, thus the effects of cigarette smoking on these two molecules are of interest.

The Nutrition Examination Survey (NHANES III) cross-sectional study conducted in the United States^13^ found that low intake of vitamin C, an essential dietary requirement for humans, is a risk factor for periodontal disease. Furthermore, gingival bleeding in vitamin C-deficient patients has been shown to be improved by vitamin C supplementation^14,15^. The amount of vitamin C in the blood of smokers is known to be lower as compared to nonsmokers^16^, thus elucidation of the molecular basis for the effects of vitamin C on onset and progression of periodontal disease in smokers is important.

Based on results of the present study, it is suggested that CSE disrupts the barrier function of gingival epithelium via JAM1 translocation, thus allowing for penetration of bacterial virulence factors into subepithelial tissues. Additionally, they provide the molecular basis for cigarette smoking as a risk factor for periodontal disease development. In addition, vitamin C is indicated as a potential nutrient to increase the barrier function of gingival epithelium exposed to cigarette smoke.

## Results

### CSE causes loss of JAM1, but not CXADR, on surface of gingival epithelial cells

**Figure 1:**
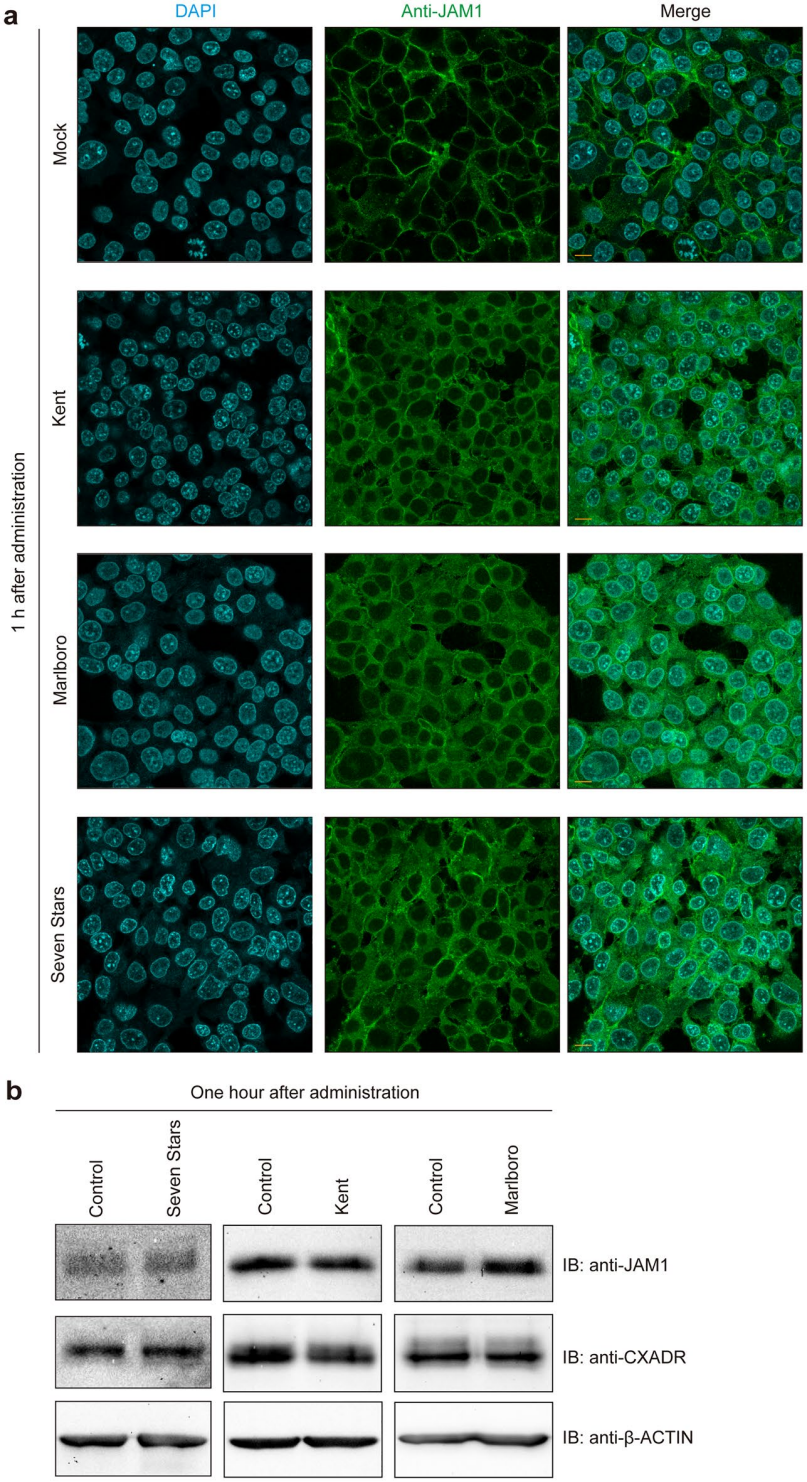
CSE causes JAM1 mislocalization in IHGE cells. (**a**) IHGE cells were exposed to CSE (Kent, Marlboro, Seven Stars) for 1 h. The cells were then fixed, stained with DAPI (cyan), mouse monoclonal anti-JAM1 (FITC; green in Fig. 1A), rabbit monoclonal anti-CXADR (Alexa 555; magenta, see also in Supplementary Figure 1), and analyzed using confocal microscopy. The same area as in Supplementary Figure 1 was photographed with only DAPI and FITC wavelengths. Scale bars, 10 µm. (**b**) IHGE cells were exposed to CSE for 1 h, then analyzed using immunoblotting with the indicated antibodies. β-ACTIN was used as a loading control. IB, immunoblot. Full-length blots are shown in Supplementary Figure 7.

The effects of CSE on JAM1 and CXADR distribution were initially examined. At 1 h after administration of CSE derived from Kent, Marlboro, or Seven Stars brand cigarettes, JAM1 was found to have disappeared from the cellular surface (Fig. 1a), while negligible effects were observed in regard to CXADR localization (Supplementry Figure 1). In addition, the findings confirmed that JAM1 and CXADR levels were not altered by CSE (Fig. 1b). The same trends were confirmed in examinations of primary gingival epithelial cells (Supplementary Figure 2). Hence, CSE affects JAM1 localization, but not the level of that protein.

**Figure 2:**
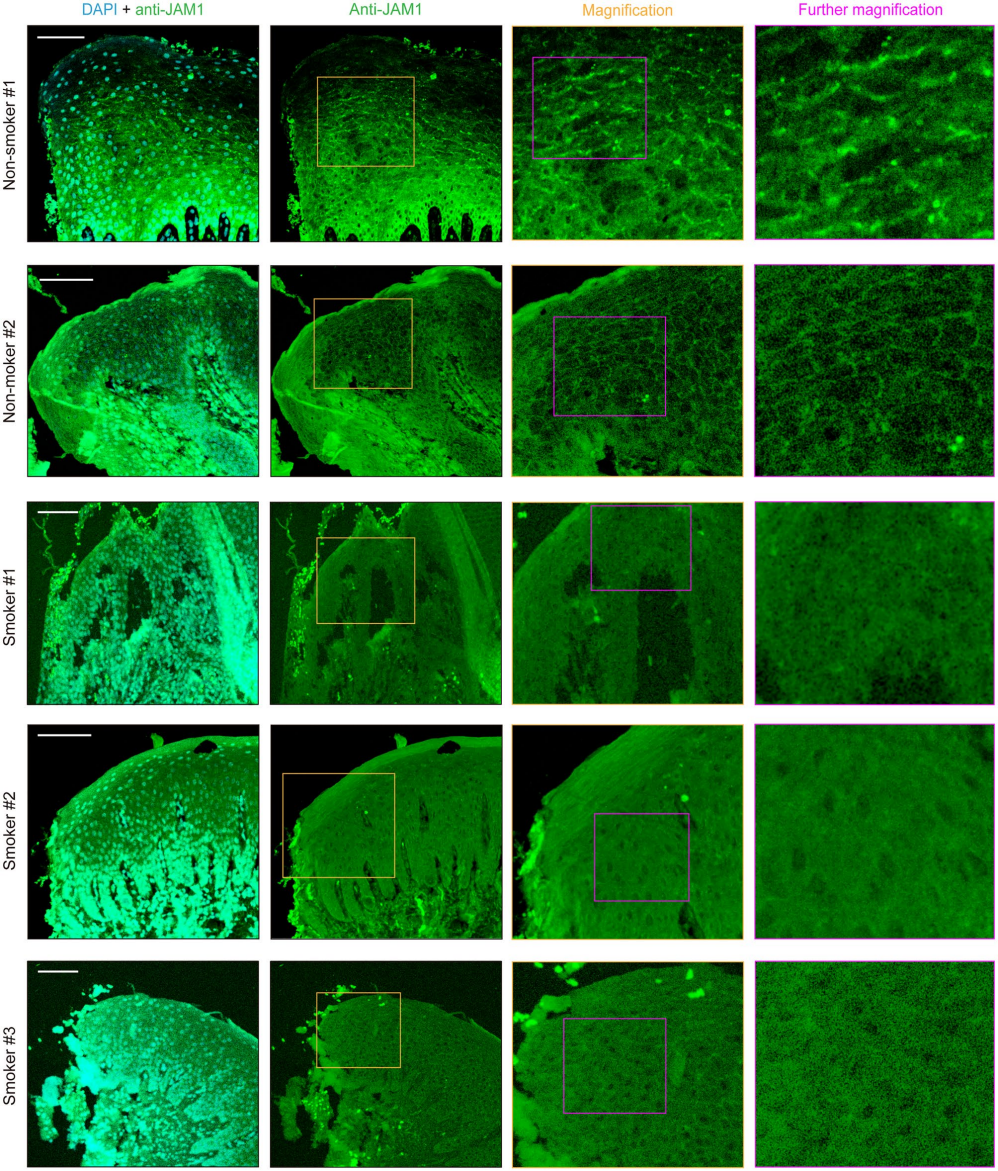
CSE causes JAM1 mislocalization in gingival epithelium. Human gingival tissues from non-smokers and smokers were fixed and stained with DAPI (cyan) and rabbit monoclonal anti-JAM1 (Alexa Fluor 635; green), then analyzed using confocal microscopy. The orange square areas in the Anti-JAM1 column are shown magnified in the Magnification column, while the Further magnification column shows magnification of the magenta square in that column. Scale bars, 100 μm.

The distribution of JAM1 in human gingival tissues of smoker and non-smoker subjects was also examined. In gingival epithelium specimens obtained from the non-smokers, JAM1 showed a grid-liked pattern, while scattered localization was noted in specimens from the smokers (Fig. 2). Even the specimen obtained from Smoker #1, who was without bleeding on probing and showed periodontal pockets ≥ 4 mm, JAM1 had lost its original localization. These results prompted us to examine whether cigarette smoking diminishes intercellular JAM1 localization in human gingival epithelia.

### JAM1 translocation from cell surface to EGFR-positive endosomes induced by CSE

**Figure 3:**
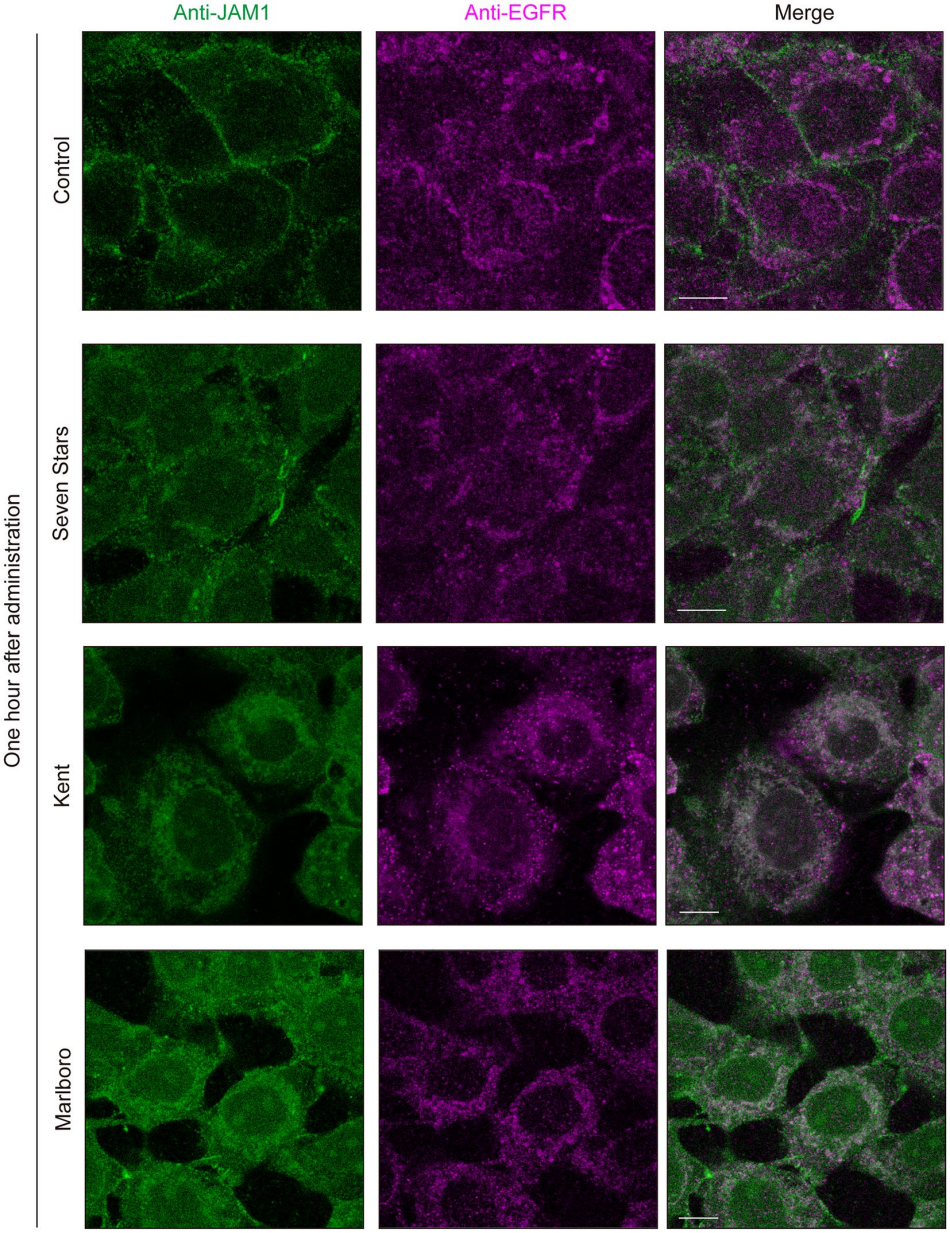
CSE induces translocation of JAM1 to EGFR-positive endosomes in IHGE cells. IHGE cells were exposed to CSE for 1 h, then fixed, stained with mouse monoclonal anti-JAM1 (FITC; green) and anti-EGFR (Alexa Fluor 635; magenta), and analyzed using confocal microscopy. Scale bars, 10 µm.

**Figure 4:**
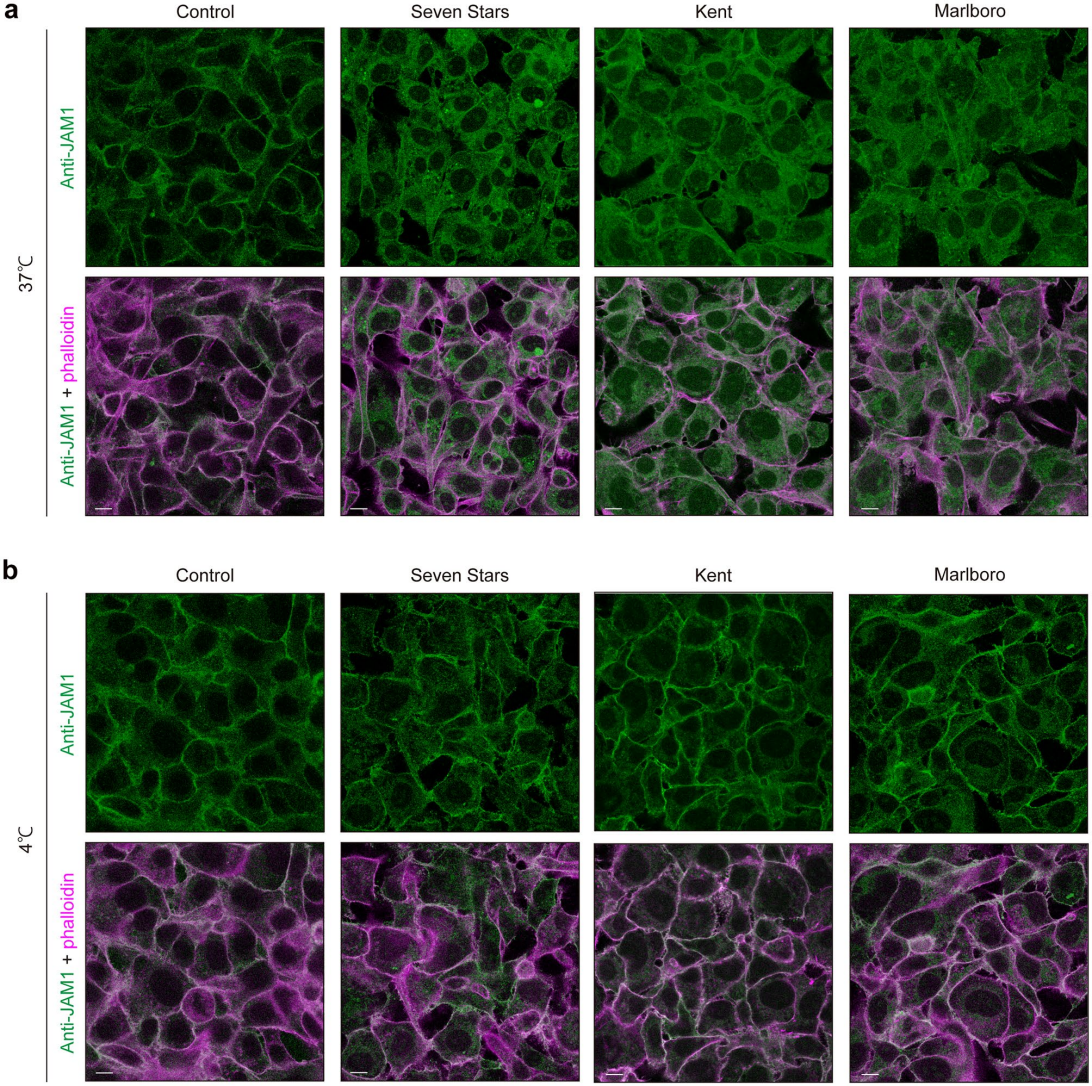
Confocal microscopic images of JAM1 in CSE-treated IHGE cells at different temperatures. IHGE cells were kept at (**a**) 37 °C or (**b**) 4 °C for 30 min, then exposed to CSE for 1 h. Thereafter, the cells were fixed, stained with mouse monoclonal anti-JAM1 (FITC; green) and Alexa Fluor 633-conjugated phalloidin (magenta), and analyzed using confocal microscopy. Scale bars, 10 µm.

We previously showed that JAM1 is transported to the plasma membrane via an endomembrane system^10^, which consists of different membranes suspended in cytoplasm within a eukaryotic cell. It was thus speculated that CSE induces JAM1 translocation to intracellular organelles. Following CSE administration, IHGE cells were stained with anti-EGFR as a marker for the endocytosis pathway^17^. At 1 h after CSE administration, JAM1 was clearly found located in EGFR-positive endosomes (Fig. 3). In general, endocytosis does not occur below 10 °C^18^. To confirm whether CSE induces translocation of JAM1 from the plasma membrane via the endocytic pathway, IHGE cells were treated with CSE at 4 °C, though JAM1 localization remained on the plasma membrane for up to 1 h (Fig. 4). These findings were also confirmed in primary gingival epithelial cells (Supplementary Figure 3). Additionally, fractionation of IHGE cells showed that JAM1 and CXADR could be detected in membrane compartments including plasma membrane and endosomes, but not in cytosol compartments, in either the CSE-added or non-added cells (Supplementary Figure 4a). Isolation of plasma membrane fraction of IHGE cells showed that CSE treatment decreased JAM1 levels, but not of CXADR, in the plasma membrane fraction (Supplementary Figure 4b). Together, these results suggest that JAM1 selectively internalized in the plasma membrane is induced by CSE to translocate to gingival epithelial cells.

### CSE induces penetration of LPS and PGN into gingival epithelium

**Figure 5:**
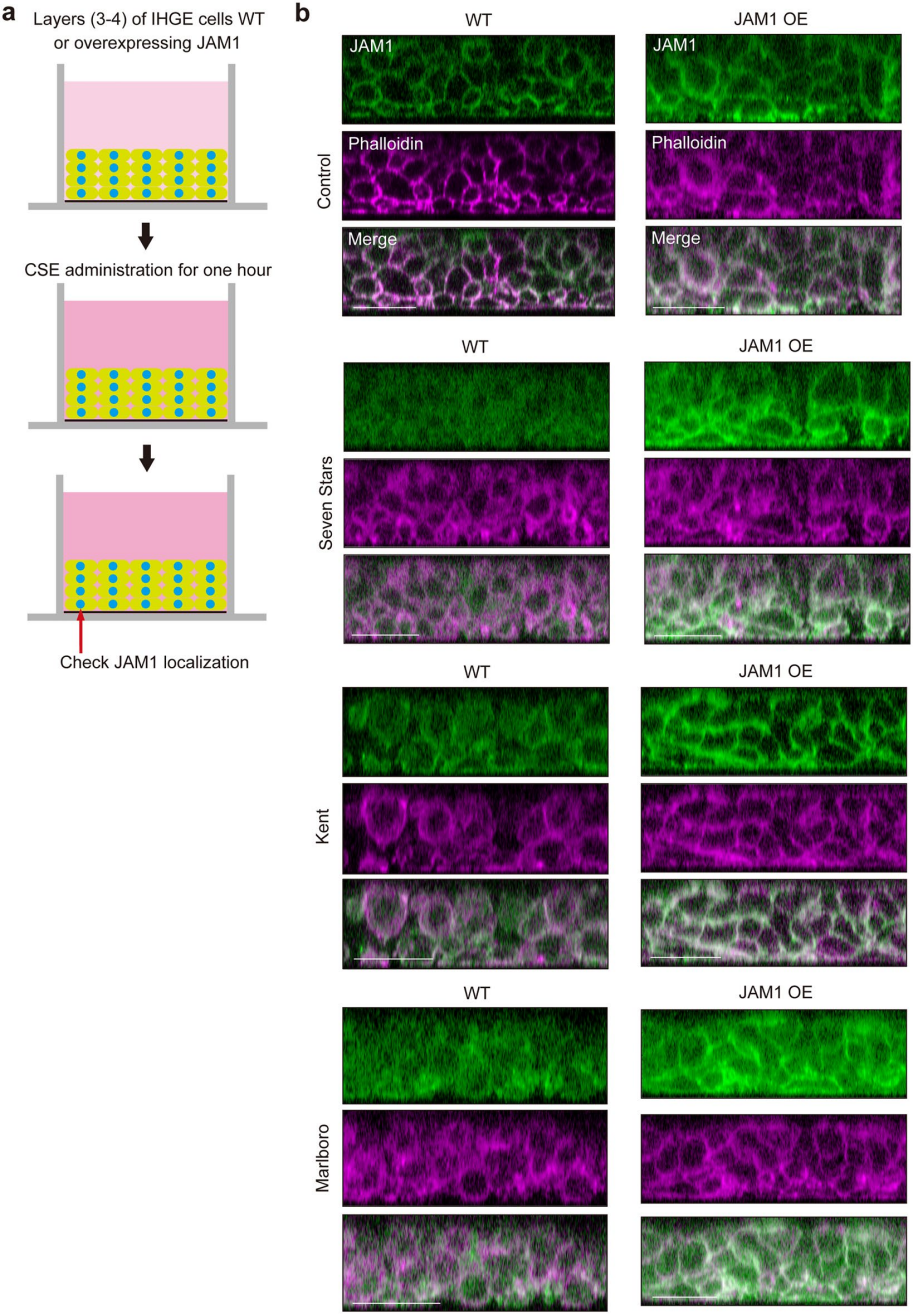
Confocal microscopic images of JAM1 in 3D-tissue model of IHGE cells exposed to CSE. (**a**) Schematic illustration and (**b**) confocal microscopic cross-sectional images of 3D-tissue model of IHGE cells. Gingival epithelial tissues on coverslips were exposed to CSE for 1 h, then fixed, stained with mouse monoclonal anti-JAM1 (FITC; green) and Alexa Fluor 633-conjugated phalloidin (magenta), and analyzed using confocal microscopy. Scale bars, 30 µm. OE: overexpression.

To assess the effects of CSE on JAM1 localization in deeper epithelium, a 3D-tissue model of gingival epithelium was generated using a cell-accumulation technique^19^ (Fig. 5a). JAM1 proteins were found localized in phalloidin-stained plasma membranes in the model without CSE (Fig. 5b). At 1 h after CSE administration, JAM1 was found to have translocated from cell surfaces to intracellular space in the tissues up to 3–4 layers below the surface, which was effectively compensated by JAM1 overexpression. These results suggest that CSE causes loss of surface JAM1 in human gingival epithelial tissues.

**Figure 6:**
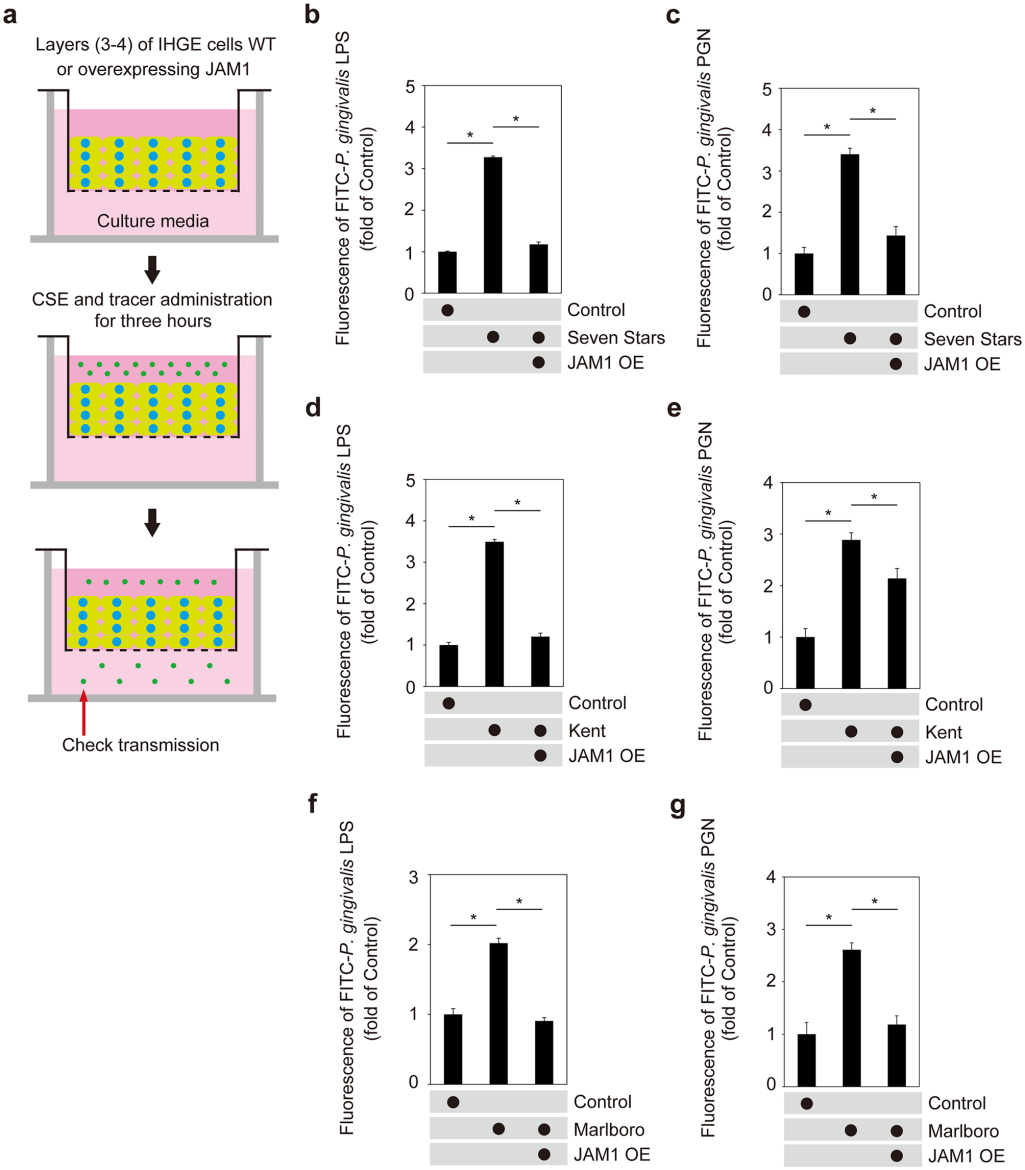
Effects of CSE on permeability of gingival epithelium to LPS and PGN. (**a**) Schematic illustration of three-dimensional culture of IHGE cells. (**b**–**g**) Permeability of gingival epithelial tissues (WT or with JAM1 overexpression) to FITC–*P. gingivalis* LPS (**b**, **d**, **f**) and FITC–*P. gingivalis* PGN (**c**, **e**, **g**) following exposure to CSE. Three-dimensional tissues in culture inserts with or without CSE in the upper compartment were treated with an FITC-labeled tracer. Following 3 h of incubation, transmission of the tracer from the upper to lower compartment was analyzed using spectrometry. Values are expressed as fold change relative to the control (WT cells) shown as the mean ± SD of eight technical replicates. *p < 0.05, one-trailed *t* test (closed testing procedure).

It was previously reported that cigarette smoke facilitates allergen penetration through human bronchial epithelial cell monolayers^4^. Bronchial epithelium has a pseudostratified ciliated columnar structure, whereas gingival epithelium has a stratified squamous form, thus we attempted to clarify the biological implications of loss of JAM1 on the gingival epithelium surface induced by CSE. 3D-tissue models of IHGE wild type (WT) cells or those overexpressing JAM1 were generated, then permeability assays using fluorescein isothiocyanate (FITC)-labeled *P. gingivalis* LPS and PGN were performed (Fig. 6a). At 3 h after administration, permeability to both LPS (Fig. 6b,d,f) and PGN (Fig. 6c,e,g) in the examined tissues was significantly increased by CSEs. In contrast, of JAM1 overexpression effectively compensated for its translocation even after exposure to CSE. These results suggest that JAM1 translocation by CSE allows for penetration of LPS and PGN into human gingival epithelium.

### CSE inhibition of cellular migration and proliferation dependent on JAM1

**Figure 7:**
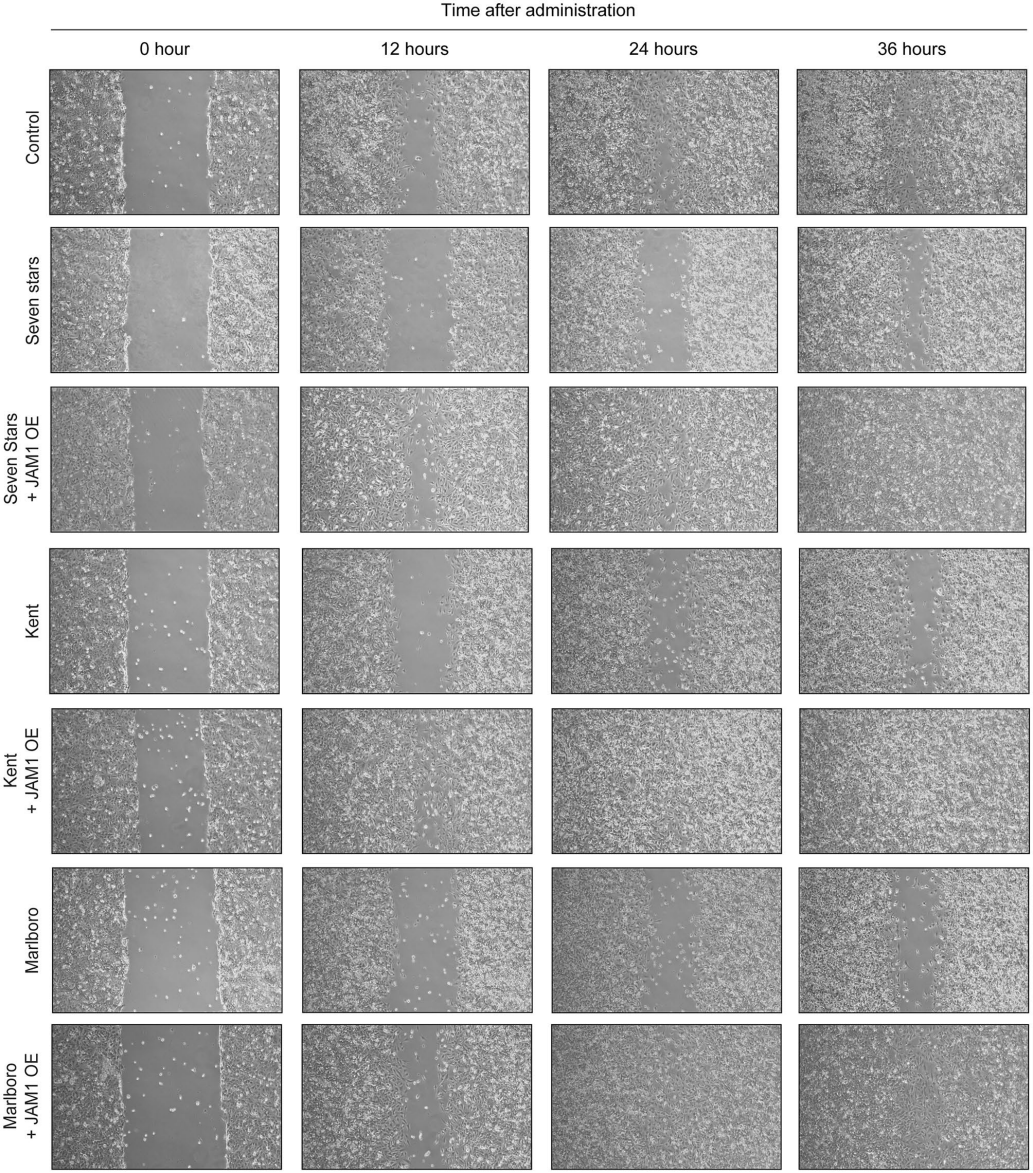
Effects of CSE on migration of IHGE cells. Confluent monolayers of IHGE cells (WT or with JAM1 overexpression) were scratched with a pipette tip, then treated with CSE for the indicated time period. The cells were then analyzed using phase contrast microscopy.

Cellular migration and proliferation are critical functions for wound healing and tissue regeneration of periodontal tissues destroyed by periodontal pathogens^20^. JAM1 has been reported to regulate the migration of human umbilical vein endothelial cells^21^. Thus, whether CSE inhibits cellular migration and/or proliferation was examined using an in vitro wound healing assay. After IHGE cell monolayers were scratched, the cells proliferated and migrated to fill the scratched areas in a time-dependent manner (Fig. 7), while scratch closure in IHGE cells was found to be inhibited at 12, 24, and 36 h after CSE administration. On other hand, such inhibitory effects were not clearly seen in IHGE cells overexpressing JAM1, suggesting its involvement in inhibition of cellular migration and proliferation by CSE. Collectively, the present morphological, permeability, and scratch assay results suggest that CSE inhibits JAM1 function, and increases periodontal tissue vulnerability.

### Vitamin C induces JAM1 expression in gingival epithelial cells

**Figure 8:**
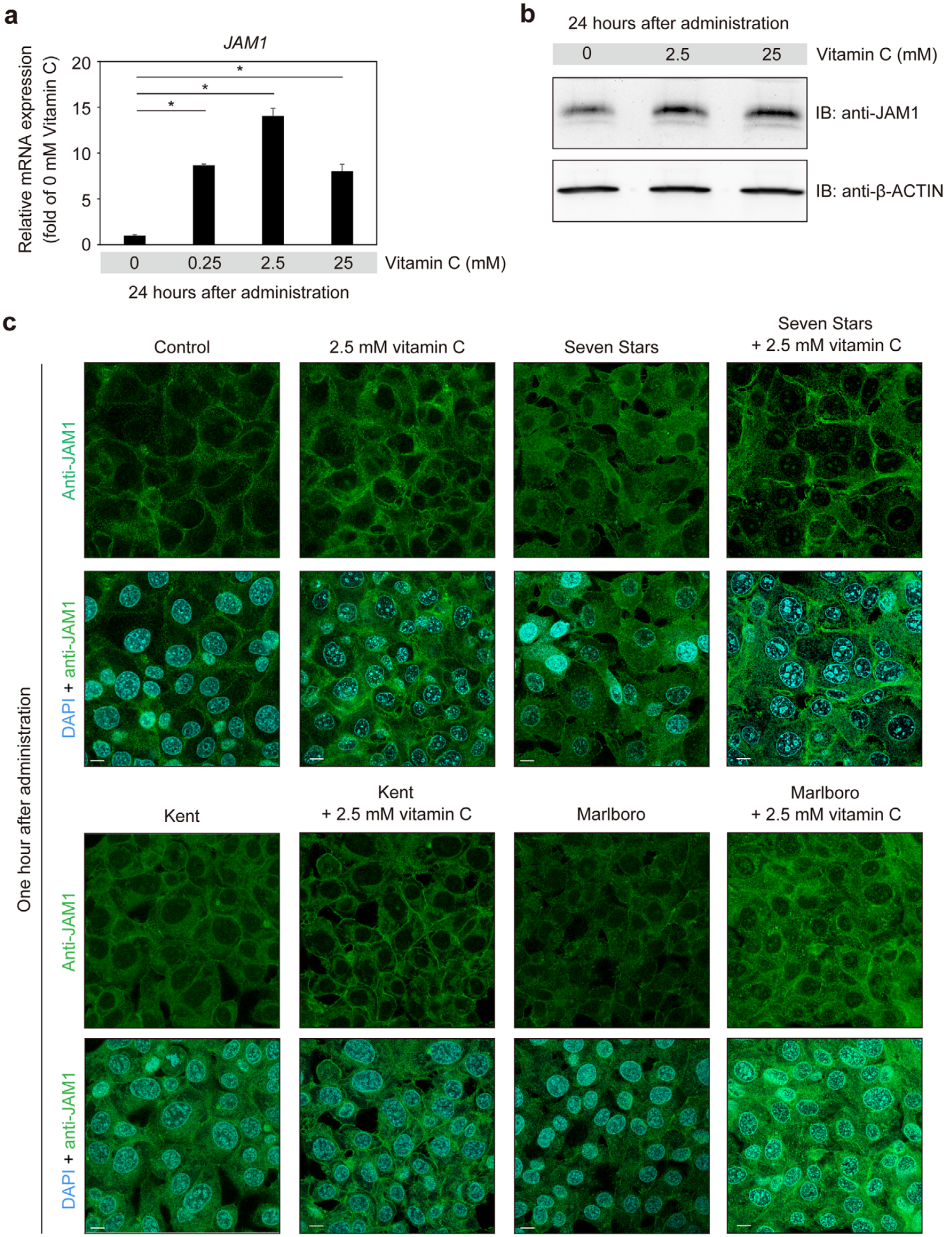
Vitamin C induces JAM1 expression in IHGE cells. (**a**) Relative levels of JAM1 mRNA expression in vitamin C-treated IHGE cells. IHGE cells were treated with vitamin C at the indicated concentration for 24 h, then sampled for a qRT-PCR assay. Results are expressed as fold change relative to no administration with five technical replicates. The significance of differences was evaluated using a two-tailed *t* test. (**b**) IHGE cells were treated with vitamin C at the indicted concentration. Following 24 h of incubation, cells were analyzed by immunoblotting with the indicated antibodies. Full-length blots are shown in Supplementary Figure 7. (**c**) IHGE cells were treated with 2.5 mM vitamin C and incubated for 1 h, then treated with the combination of CSE and vitamin C, and incubated for 1 h. The cells were then fixed, stained with DAPI (cyan) and mouse monoclonal anti-JAM1 (FITC; green), and analyzed using confocal microscopy. Scale bars, 10 μm.

While an inverse association of smoking with vitamin C in serum has been reported^22^, no evidence of a relationship of vitamin C and cigarette smoke-exposed epithelial barrier function has been presented. Thus, the effects of vitamin C on the permeability of gingival epithelium were assessed. Gene and protein expressions of JAM1 were significantly increased in IHGE cells following administration of vitamin C (Fig. 8a,b). Similarly, vitamin C increased localization of cellular-surface JAM1 in IHGE cells (Fig. 8c). Next, IHGE cells were treated with vitamin C in combination with CSE. As shown in Fig. 8c, cellular-surface JAM1 was clearly localized even in the presence of CSE in a manner similar to the control, suggesting that vitamin C inhibits the effects of CSE.

### Vitamin C prevents LPS and PGN penetration into CSE-treated gingival epithelium

**Figure 9:**
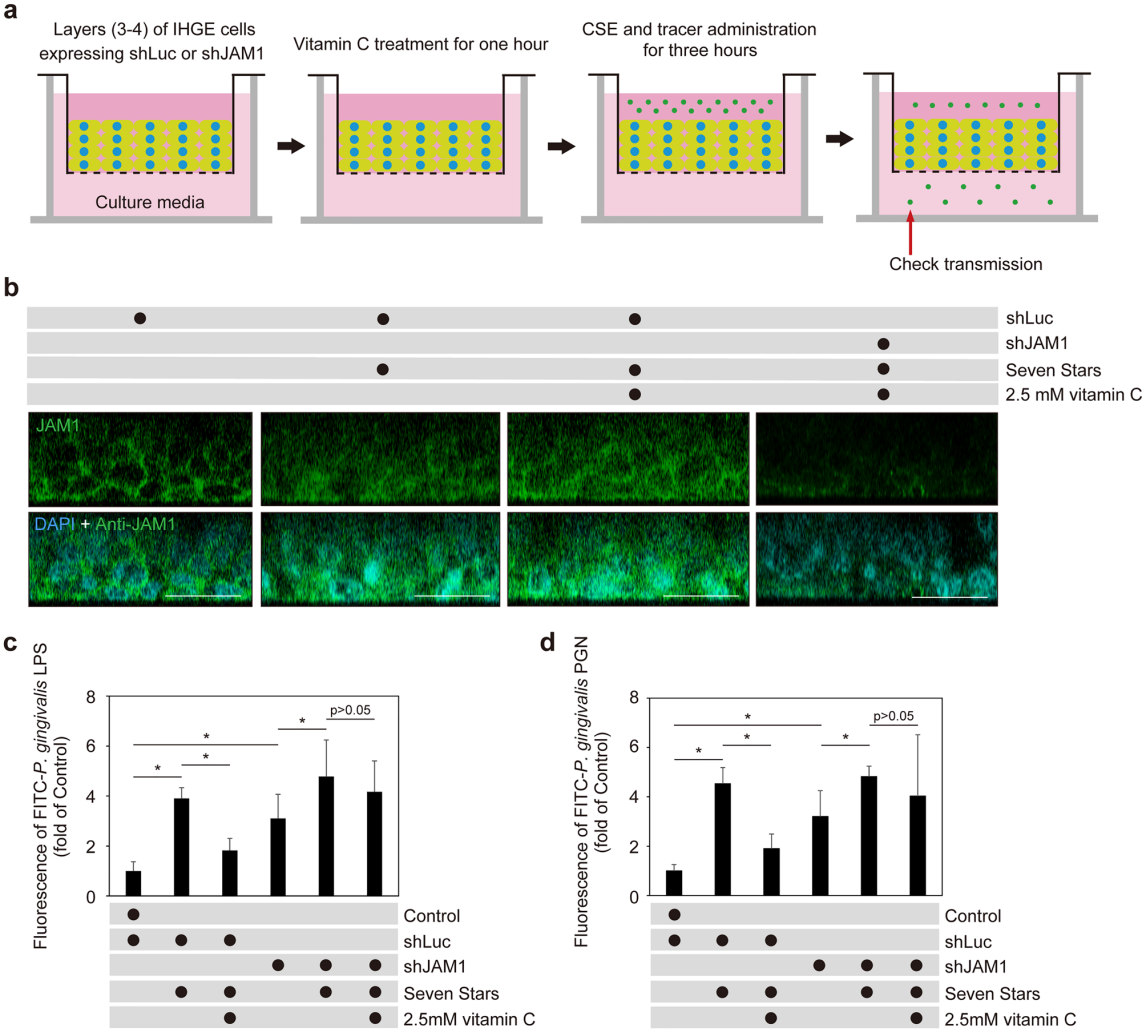
JAM1 involved in vitamin C-induced restoration of CSE-disturbed barrier function against LPS and PGN in human gingival epithelium. (**a**) Schematic image of culture insert system and (**b**) representative confocal microscopic cross-sectional images of three-dimensional culture of IHGE cells (DAPI; cyan, mouse monoclonal anti-JAM1: FITC; green). A multilayer of IHGE cells expressing shLuc or shJAM1 was cultured in the upper compartment, with vitamin C administered to samples in the upper compartments. Following 1 h of incubation, Seven Stars SCE and fluorescent tracers were administered, and the tissues were cultured for 3 h, after which culture medium from the lower compartment was analyzed using spectrometry. (**c**, **d**) Permeability of gingival epithelial tissues to FITC-*P. gingivalis* LPS (**c**) or PGN (**d**) expressing indicated shRNA, with or without addition of Seven Stars CSE and vitamin C. Three-dimensional tissues in culture inserts were treated with vitamin C and incubated for 1 h. Seven Stars CSE and FITC-labeled tracers were then administered to tissues in the upper compartment, and those were cultured for 3 h, after which transmission of the tracer from the upper to lower compartment was analyzed using spectrometry. Results are expressed as fold change relative to untreated shLuc-expressing cells. Values are shown as the mean ± SD of eight technical replicates. *p < 0.05, one-tailed *t* test (closed testing procedure).

To clarify the contribution of JAM1 to the compensating effects of vitamin C in gingival epithelium treated with CSE, IHGE cells expressing short hairpin RNA (shRNA) against JAM1 (shJAM1) for depletion of JAM1 expression were generated^11^. 3D-tissue models generated using IHGE cells expressing shRNA against *Renilla* luciferase (shLuc; as a control) or shJAM1 were used to analyze the localization of JAM1 in gingival epithelial tissues (Fig. 9a). As shown in Fig. 9b, the effect of CSE on JAM1 was sufficiently compensated by vitamin C, while conversely that was abrogated by JAM1 knockdown. Permeability assay findings also indicated that vitamin C decreased permeation of *P. gingivalis* LPS (Fig. 9c) and PGN (Fig. 9d) in tissues expressing shLuc, while that rescue was abrogated by JAM1 knockdown. Based on these results, it was concluded that JAM1 is involved in the compensating effect of vitamin C to counter the effects of CSE in human gingival epithelial tissues.

## Discussion

Results of the present study indicated that CSE causes translocation of JAM1 from the host cell membrane surface to cytoplasm, resulting in increased penetration by LPS and PGN of the gingival epithelial barrier. Additionally, vitamin C was found to induce JAM1 expression on the surface of gingival epithelial cells in CSE-treated tissues, resulting in rescue of barrier functions against LPS and PGN. Cigarette smoke contains more than 7000 chemicals, of which at least 250 are known to be harmful^23^. For example, nicotine is reported to constrict blood vessels and decrease blood flow^24^, and tar is known to subvert immune systems^25^. Furthermore, non-pathogenic inflammatory triggers, including LPS and PGN, and cigarette smoke components can synergistically exacerbate a periodontal disease condition. To the best of our knowledge, these are the first reported findings of the molecular mechanism of barrier dysfunction in gingival epithelium caused by cigarette smoking.

Results obtained with the present in vitro model and gingival tissue samples from smokers showed a characteristic similar to that seen with *P. gingivalis*, in that CSE disturbed JAM1 localization in deeper epithelium (Fig. 2). Thus, in regard to dysfunction of JAM1 in gingival epithelium, smokers have the same condition seen with individuals with a *P. gingivalis* infection. These two periodontal risk factors show the same phenotypic alteration in gingival epithelium in the form of dysfunction of tight junction-associated proteins, suggesting that the barrier function of gingival epithelial tissues is closely involved in periodontal disease pathogenesis.

Although the present study sought to determine the association between CSE and gingival epithelial tissues, it may be worthwhile to examine JAM1 in other infectious diseases that develop in organs that have direct contact with tobacco smoke. It is known that exposure to tobacco smoke is a substantial risk factor for many acute respiratory bacterial infections, including pneumonia^26^ and meningococcal disease^27^, as *Streptococcus pneumoniae*, a pneumonia pathogen, downregulates the tight junction proteins Claudin-7 and -10^28^, while *Neisseria meningitidis*, which causes meningococcal pneumonia, disrupts occludin, another tight junction protein^29^. Generally, tissue invasion by LPS induces neutrophils in the lungs and causes lung inflammation. However, the biological effects of these bacteria at the molecular level on the permeability of bacterial toxins are unknown. To clarify the causal link between cigarette smoking and increased risk of pneumonia severity, investigation of epithelial barrier function related to JAM1 in lung epithelium is potentially important.

A review of previous studies found that smokers are more likely to develop severe disease conditions related to influenza^30^ or coronavirus disease 2019 (COVID-19) caused by the severe acute respiratory syndrome coronavirus 2 (SARS-CoV-2)^31^. Infection with the influenza virus was shown to decrease Claudin-5, a tight junction protein, in vitro^32^, while that protein has also been reported to be decreased in fetal lung sections infected with COVID-19^33^. In reconstructed human bronchial epithelium specimens, SARS-CoV-2 infection was reported to disturb ZO-1 localization^34^, while JAM1 has been shown to form a complex with ZO-1^35^. These results indicated that a dysfunctional epithelial barrier is responsible for the spread of organisms causative of influenza and COVID19 disease in tissues. Hence, the causal relationship of viral infections with tight junction-associated proteins shows the need for further investigation.

The present results showed that CSE affects JAM1 but not CXADR. JAM1 and CXADR are both JAM family proteins with similar structures, though there are some slight structural differences. For example, JAM1 cytoplasmic tails are shorter than those of CXADR and the five C-terminal residues of the JAM1 protein possess a class II PDZ domain binding motif, while CXADR possesses class I PDZ domain binding motifs in cytoplasmic tails. In consideration of the present results, CSE may specifically target JAM1-specific sites in cytoplasmic tails. Of note, not all IHGE cells with JAM1 overexpression were disrupted by CSE (Supplementary Figure 5), suggesting that tobacco components are not directly involved with JAM1 molecules, but rather that a bottleneck, such as receptors or mediators against tobacco ingredients, is formed in the process of CSE-induced JAM1 endocytosis.

Cells internalize plasma membrane proteins by endocytosis, with removal of the membrane from the cell surface balanced by recycling pathways that return some of the endocytosed proteins back to the plasma membrane^36^. Essentially, endocytosed proteins become degraded, though endocytosed JAM1 after CSE treatment did not appear to be degraded (Fig. 1b). Non-degraded proteins may be transported to the plasma membrane under a normal condition, and that did not seem to occur with JAM1 after CSE treatment in vitro (Fig. 1a) or in vivo (Fig. 2). These findings suggest that CSE not only induces JAM1 endocytosis but also stabilizes its subsequent intracellular transport to the plasma membrane. Since the recycling pathway is involved in maintenance of cellular homeostasis, the effect of tobacco smoke on membrane traffic is an interesting subject for further study.

Smoking cessation is one of the most important ways to eliminate the risk of periodontal disease development and progression^23,37^. To motivate smokers to quit, explanation of the harmful effects of smoking can be effective, because health issues are of primary concern to many people. We believe that providing information regarding not only smoking-related health risks but also details of the molecular mechanism of the etiology of tobacco-induced periodontal diseases, as well as other diseases noted above will help patients change their behavior.

## Materials and methods

### Immunohistochemistry

All human subjects who participated provided informed consent to the study protocol, which was reviewed and approved by the ethics committee of Osaka University Graduate School of Dentistry (R2-E8-2). The methods were performed in accordance with the ethical guidelines for life sciences and medical research involving human subjects established by the Ministry of Health, Labour, and Welfare in Japan. Gingival samples were obtained from two non-smokers and three smokers, with details regarding age, gender, tooth type, pocket depth, bleeding on probing, medical history, and reason for tooth extraction for the subjects shown in Supplementary Table 1. Each was negative for human immunodeficiency virus, hepatitis B virus, human hepatitis C virus, and diabetes, and none were taking medication possibly associated with gingival hyperplasia (e.g., nifedipine, cyclosporine, phenytoin) at the time of the study.

Immunohistochemistry was performed as previously described^38^. Briefly, samples were fixed with 4% paraformaldehyde/phosphate-buffered saline (PFA/PBS) overnight at 4 °C, then embedded in paraffin and cut into 5-μm sections, which were then deparaffinized and rehydrated. Antigen retrieval was performed by heating sections in antigen retrieval buffer (pH 9) (Nichirei Biosciences) using an electric thermo pot (NC-BJ221; Panasonic) for 40 min at 98 °C, followed by cooling to room temperature. After blocking with 1% bovine serum albumin, sections were incubated with an anti-JAM1 antibody (1:100 dilution, HPA-061700; Atlas Antibodies) at 4 °C for 15 h, washed thrice with TBST, then incubated with Alexa Fluor 635-conjugated anti-rabbit immunoglobulin G (1:500 dilution, A-31576; Thermo Fisher Scientific). Nucleus staining was performed with 4’,6-diamidino-2-phenylindole (DAPI; Invitrogen). Sample immunoreactivity was analyzed using a Leica TCS SP8 confocal microscope (Leica Microsystems).

### Preparation of CSE

CSE was prepared as previously described^39^. Briefly, three types of commercial cigarettes (Seven Stars Box: Japan Tobacco Inc; Kent 1 100S Box: British American Tobacco Japan; Marlboro: Phillip Morris) were lit and the smoke bubbled into 10 ml of Humedia KG-2 (Kurabo) with a polypropylene tube (inner diameter 8 mm) and plastic syringe (20 ml) until the cigarette burned out, using the equipment shown in Supplementary Figure 6. The tar and nicotine contents of each brand of tested are shown in Supplementary Table 2. Each CSE sample was extracted from a single cigarette and immediately used. For immunoblotting, immunocytochemistry, and epithelial barrier function assays, CSE at 10 ml, 1 ml, and 500 µl, respectively, per well was used. For scratch wound-healing assays, 2 ml of CSE diluted 1:25 in Humedia KG2 was used.

### Cell culture and construction of 3D-gingival epithelial tissues

IHGE cells (epi 4, kindly provided by Shinya Murakami, Osaka University)^40^ were maintained in Humedia KG-2 (Kurabo). The primary human gingival epithelial cells (HGEPp, pooled [3 or more donors], CELLnTEC; https://cellntec.com/wp-content/uploads/pdf/HGEPp-1.pdf) were grown in Humedia KG2 (containing a final concentration of 0.5 mg/mL hydrocortisone, 10 mg/mL insulin, 0.4% v/v bovine pituitary extract, 0.1 ng/mL hEGF, 50 mg/mL gentamycin, and 50 ng/mL amphotericin B, Kurabo) using the cell culture plate (100 mm, 3020–100, IWAKI). HGEPp cells were passaged by trypsin (32777–44, Nacalai Tesque) and used in the experiments at passages 1 to 3, in the same way as previously described^40^. 3D-gingival epithelial tissues were constructed as previously described^10^. After 36 h of incubation, 3D tissues were subjected to CSE experiments, with findings obtained with confocal microscopic analysis or a permeability assay. IHGE cells overexpressing JAM1, shJAM1, or shLuc were generated as previously described^10^.

### Antibodies and reagents

The antibodies and reagents used in this study are shown in Supplementary Table 3.

### Immunoblotting and immunocytochemistry

Immunoblotting and immunocytochemistry were performed as previously described^10^. Immunoreactive bands were detected using Pierce ELC Western Blotting Substrate (Thermo Scientific) and ChemiDoc XRS (Bio Rad), and images were acquired using the Quantify One software package (Bio-Rad). Confocal microscopic images were acquired with a confocal laser microscope (TCS SP8; Leica Microsystems) using a 64 × oil-immersion object lens with a numerical aperture of 1.4, then analyzed using the Application Suite X software package (Leica Microsystems).

### Epithelial barrier functional assay

FITC-tracers were prepared as previously described^10^. To assess barrier function, in vitro epithelial permeability assays were performed using 12-well cell culture inserts (353180; Corning), as previously described^10^. Fluorescence intensity was determined using a 1420 ARVO X (PerkinElmer). Data were analyzed using the WorkOut Plus software package (PerkinElmer).

### Scratch wound-healing assay

Assays for in vitro wound healing were performed as previously described^41^. Briefly, IHGE cells were cultured in a six-well microplate (3810–006; Iwaki) until confluent, then the cell layers were scratched using a plastic tip (110–705C; Watson). Next, cells were incubated with CSE-containing culture media for various periods of time. Images of cells that had migrated to the scratched areas were obtained using a phase contrast microscope (Axiovert 40 C; Carl Zeiss).

### Isolation of membrane and cytosolic fractions

Fractionation of IHGE cells were performed as previously described^42^. IHGE cells in the cell culture plate (100 mm, 3020–100, IWAKI), treated with or without Seven Stars CSE for 1 h, were washed with PBS and suspended in 1 mL of homogenization buffer (3 mM imidazole [pH 7.4], 250 mM sucrose, 0.5 mM EDTA). Cells were mechanically disrupted by vigorous passage through 23- and 27-gauge needles 8 times on ice. The sample was centrifuged at 3,000 g for 15 min at 4 °C to remove unbroken cells, host nuclei and cytoskeletal components. The sample was further centrifuged at 17,400 g for 30 min at 4 °C and the supernatant was used as the cytosolic fraction. The pellet was lysed in 250 μL of lysis buffer (2 M thiourea, 7 M urea, 3% CHAPS, 1% Triton X-100) on ice for 30 min. After centrifugation at 17,400 g for 30 min at 4℃, the supernatant was used as the membrane fraction.

Isolating plasma membrane was performed as previously described^43^. IHGE cells in the cell culture plate (100 mm, 3020–100, IWAKI), treated with or without CSE for 1 h, were washed with PBS and suspended in 10 mL of ice-cold distilled water for 1 h at 4 °C to rupture cells. The plates were washed with PBS to remove the intracellular organelles, and the plasma membrane attaching to the plates was scraped and lysed in 250 μL of lysis buffer (2 M thiourea, 7 M urea, 3% CHAPS, 1% Triton X-100) on ice for 30 min. After centrifugation at 17,400 g for 30 min at 4℃, the supernatant was used as the plasma membrane fraction.

### Statistical analysis

*P* values were determined using a *t* test with the Excel software package (Microsoft), with *p* < 0.05 considered to indicate significance.

## Supplementary Information

Supplementary Information.

## Data Availability

The datasets used and analyzed for the current study are available from the corresponding author upon reasonable request.
