# Supplementary material for: Cigarette smoke extract impairs gingival epithelial barrier function
Source: Sci Rep. 2023 Jun 7;13:9228. doi: 10.1038/s41598-023-36366-z (PMC10244868; doi:10.1038/s41598-023-36366-z)

Sample	Age	Gender	Tooth	PPD (mm)	BOP	Medical history	Reason for teeth extraction	Smoking duration
Non-Smoker #1	82	Men	37 (Stump)	3	-	Angine pectoris	Vertical root fructure	None
Non-Smoker #2	42	Men	47	3	-	None	Vertical root fructure	None
Smoker #1	79	Wemen	22 (Stump)	2	-	Breast cancer	Subgingival caries	Over ten years
Smoker #2	55	Wemen	37	6	+	Depression	Actue periodontal problem	Over ten years
Smoker #3	63	Men	16	6	+	None	Actue periodontal problem	Over ten years

Supplementary Table 1. Patient status

Brand of cigarette	Source	Tar (mg)	Nicotine (mg)
Seven Stars Box	British American Tobacco Japan	1	0.1
Marlboro	JAPAN TOBACCO Inc.	14	1.2
KENT 1 100S Box	Phillip Morris	12	0.9

Supplementary Table 2. CSE ingredients

Material	Source	Cat. No.
Mouse monoclonal anti-JAM1	Sigma-Aldrich	SAB4200468
Rabbit polyclonal anti-F11R	Atlas Antibodies	HPA061700
Rabbit monoclonal anti-CXADR	Sino Biological	10799-R271
Rabbit monoclonal anti-EGFR	Cell Signaling Technology	4267
Rabbit monoclonal anti-CALNEXIN	Cell Signaling Technology	2679
Rabbit monoclonal anti-EEA1	Abcam	ab109110
Rabbit monoclonal anti-GAPDH	Cell Signaling Technology	5174
Mouse monoclonal anti-β-ACTIN	Sigma-Aldrich	M177-3
FITC-conjugated goat anti-mouse IgG	MBL	238
Alexa Fluor 488-conjugated anti-rabbit IgG	Invitrogen	A-11008
Alexa Fluor 555-conjugated anti-rabbit IgG	Abcam	ab150078
Alexa Fluor 635-conjugated anti-rabbit IgG	Invitrogen	A31576
Goat anti-mouse antibody conjugated to horseradish peroxidase	Cell Signaling Technology	7076
Goat anti-rabbit antibody conjugated to horseradish peroxidase	Cell Signaling Technology	7074
4',6-diamidino-2-phenylindole (DAPI)	Invitrogen	D1306
Alexa Fluor 633-conjugated phalloidin	Invitrogen	A12380
pMRX-IRES-Puro	Takeuchi *et al* ., 2019	-
pMRX-IRES-Puro-JAM1	Takeuchi *et al* ., 2019	-
pSINsi-hU6-shLuc	Takeuchi *et al* ., 2021	-
pSINsi-hU6-shJAM1 #508	Takeuchi *et al* ., 2021	-
Puromycin	Invitrogen	ant-pr-1
Neomycin	Invitrogen	ant-gn-1
Fluorecein Labeling Kit-NH2	Dojindo	LK-01
P. gingivalis LPS	Invitrogen	14F18-MM
P. gingivalis PGN	Takeuchi *et al* ., 2019	-
Vitamin C (L-Ascorbic acid)	Nacalai tesque	11691-62

Supplementary Table 3. Antibodies and reagents used in this study

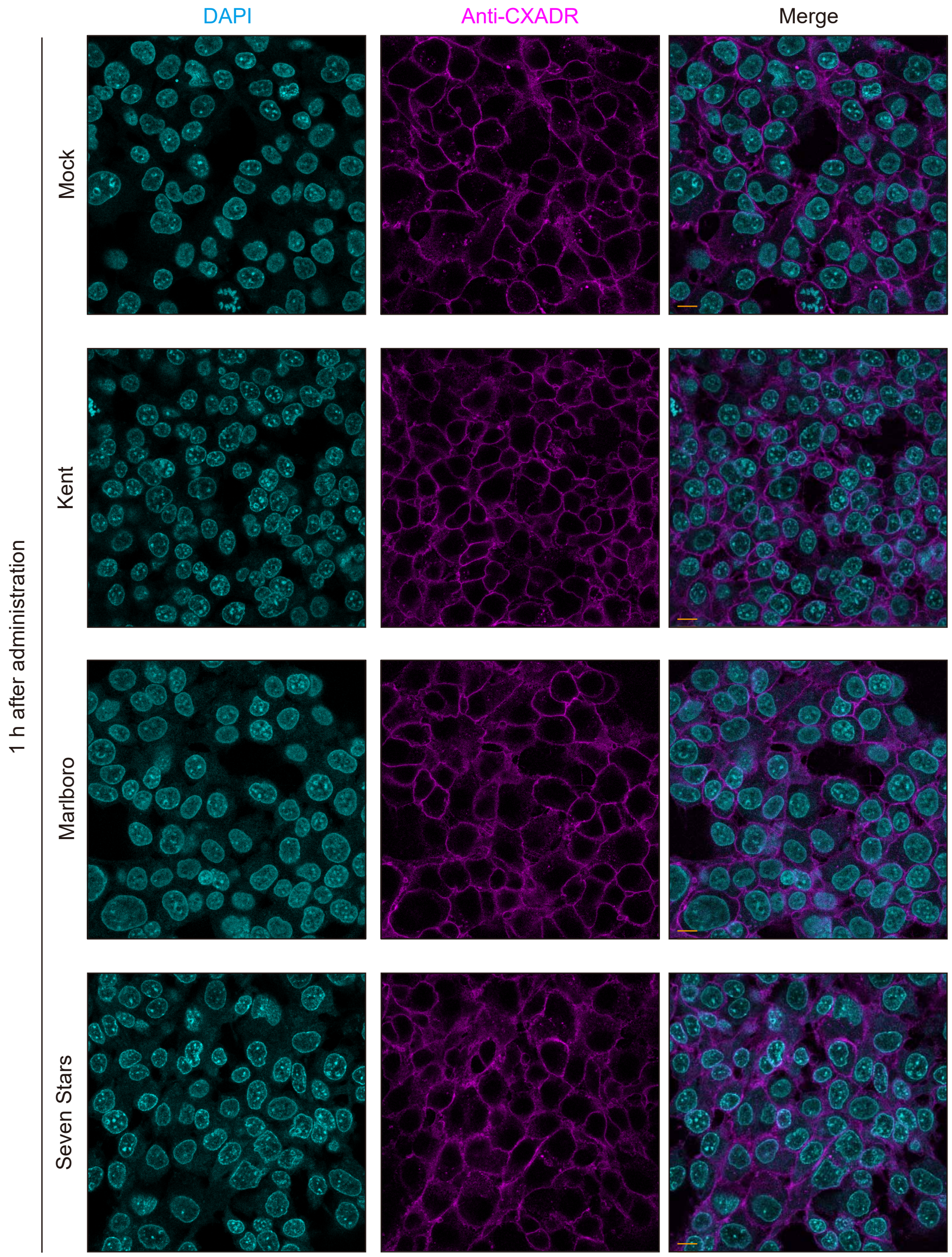

Supplementary Figure 1. Confocal microscopic images of CXADR in IHGE cells after exposure to CSE.

IHGE cells were exposed to CSE (Kent, Marlboro, Seven Stars) for 1 hour. The cells were fixed, stained with DAPI (cyan), mouse monoclonal anti-JAM1 (FITC; green, see also in Fig. 1A), rabbit monoclonal anti-CXADR (Alexa 555; magenta in Supplementary Fig. 1), then analyzed using confocal microscopy. The same area as in Figure 1A was photographed with only DAPI and Alexa 555 wavelengths. Scale bars, 10 μm.

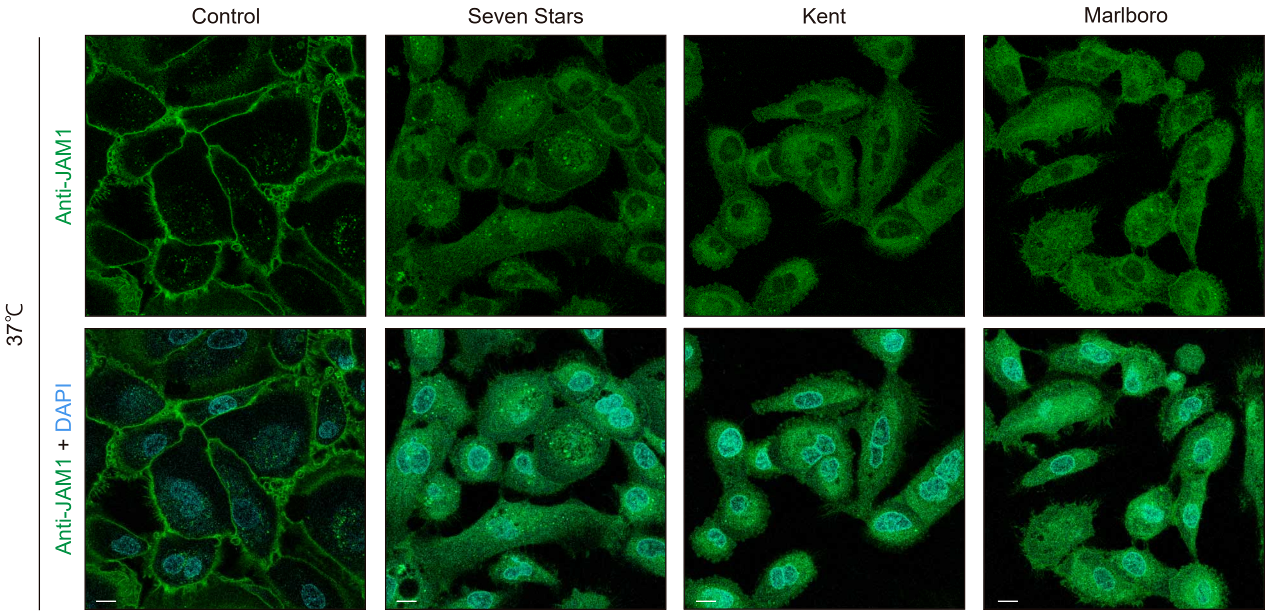

Supplementary Figure 2. Confocal microscopic images of primary human gingival epithelial cells after exposure to CSE.

Primary human gingival epithelial cells were exposed to CSE (Seven stars, Kent, Marlboro) for 1 hour. The cells were fixed, stained with DAPI (cyan) and mouse monoclonal anti-JAM1 (FITC; green), then analyzed using confocal microscopy. Scale bars, 10 μm.

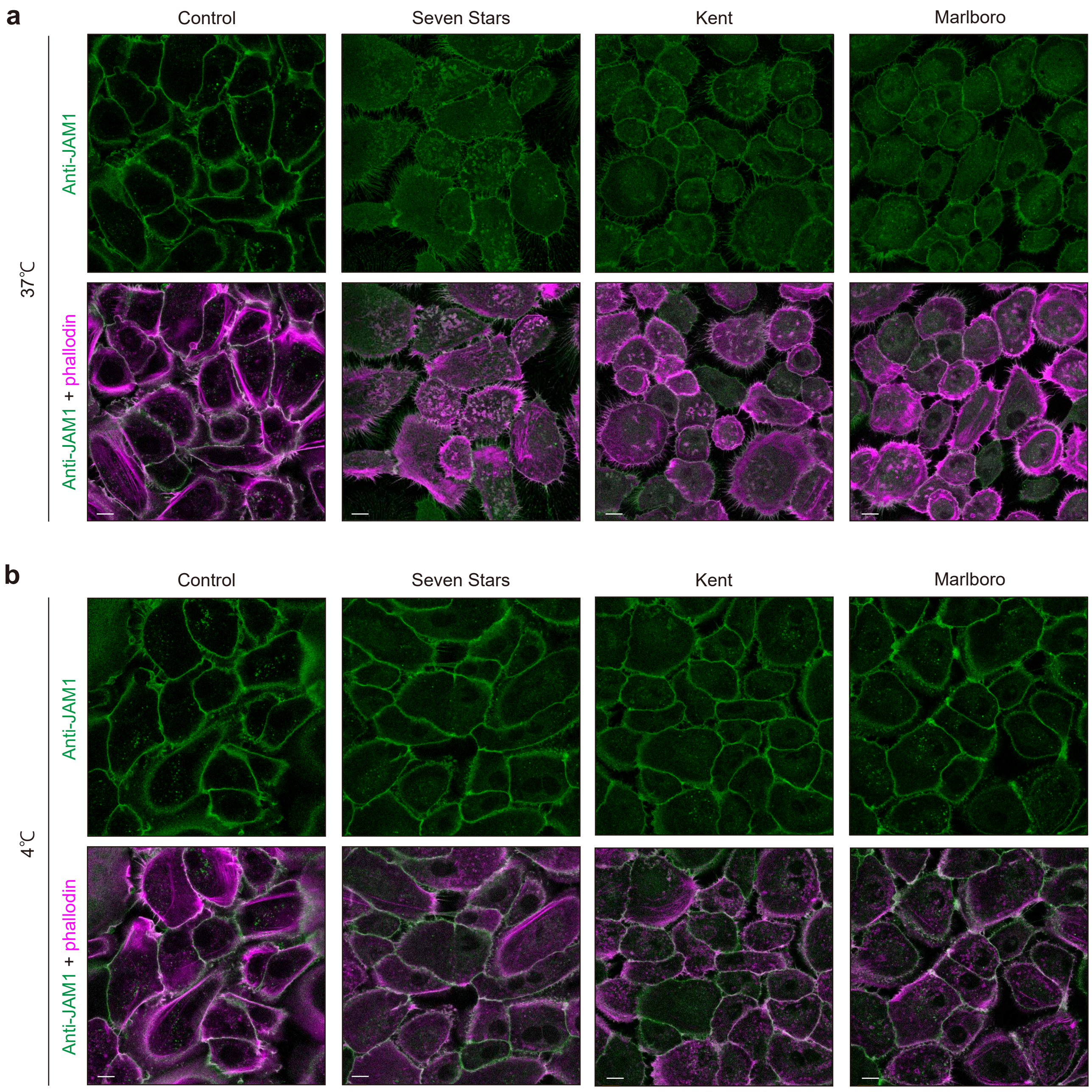

Supplementary Figure 3. Confocal microscopic images of JAM1 in CSE-treated primary human gingival epithelial cells at different temperatures.

Primary human gingival epithelial cells were kept at (a) 37˚C or (b) 4˚C for 30 minutes, then exposed to CSE for 1 hour. Thereafter, the cells were fixed, stained with mouse monoclonal anti-JAM1 (FITC; green) and Alexa Fluor 633-conjugated phalloidin (magenta), and then analyzed using confocal microscopy. Scale bars, 10 μm

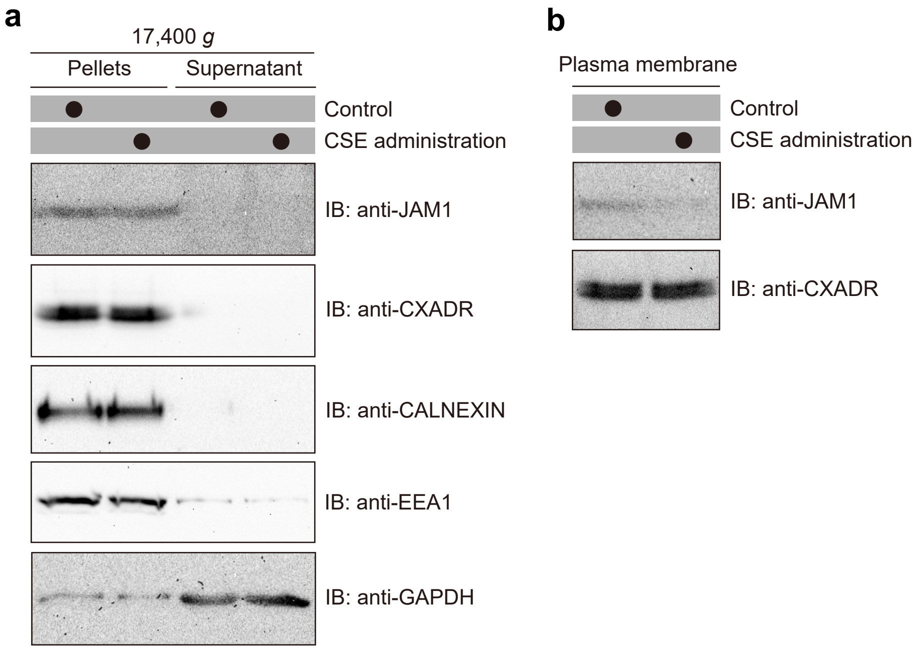

Supplementary Figure 4. Immunoblot analysis of membrane and cytosol fractions of IHGE cells after exposure to CSE.

(a,b) IHGE cells were treated with or without CSE (Seven Stars). After 1 hour, pellets (membrane fractions) and supernatant (cytosol fractions) (a), or plasma membrane fractions (b) were prepared and immunoblotted with the antibodies indicated. CALNEXIN was used as a membrane marker, early endosome antigen 1 (EEA1) was used as an endosomal marker, and glyceraldehyde-3-phosphate dehydrogenase (GAPDH) was used as a cytosol marker.

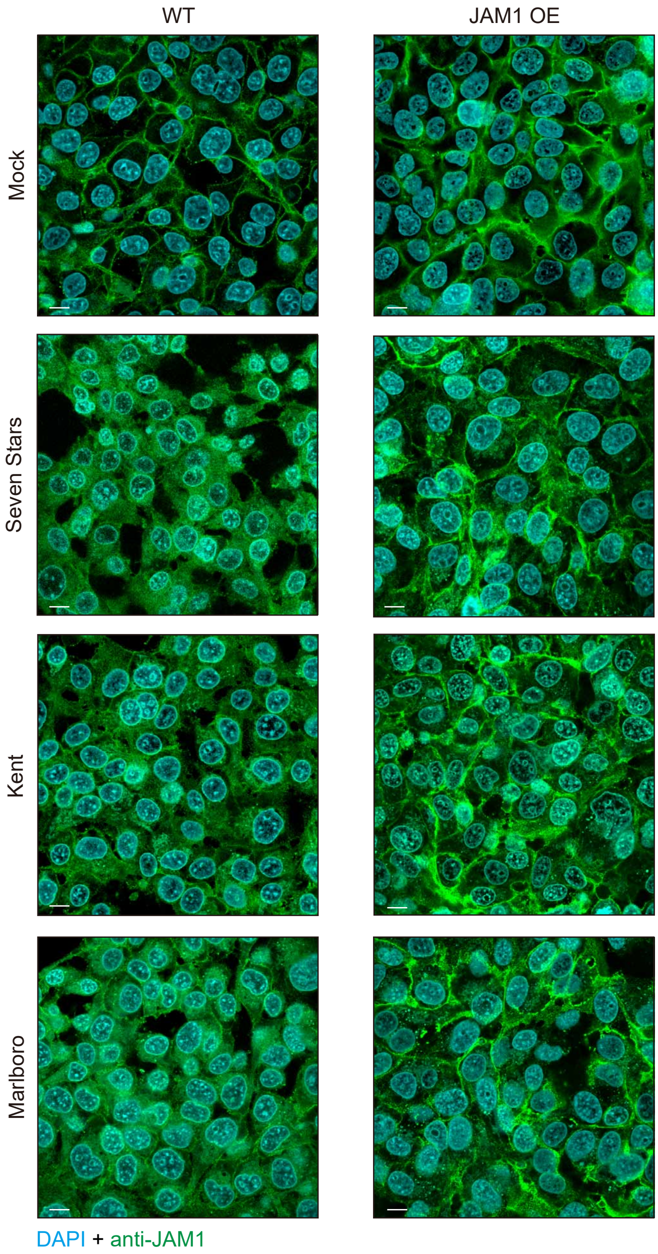

Supplementary Figure 5. Confocal microscopic images of IHGE cells overexpressing JAM1 after exposure to CSE.

IHGE cells (WT or with JAM1 overexpression) were exposed to CSE for 1 hour, then fixed, stained with DAPI (cyan) and mouse monoclonal anti-JAM1 (green), and analyzed using confocal microscopy. Scale bars, 10 μm

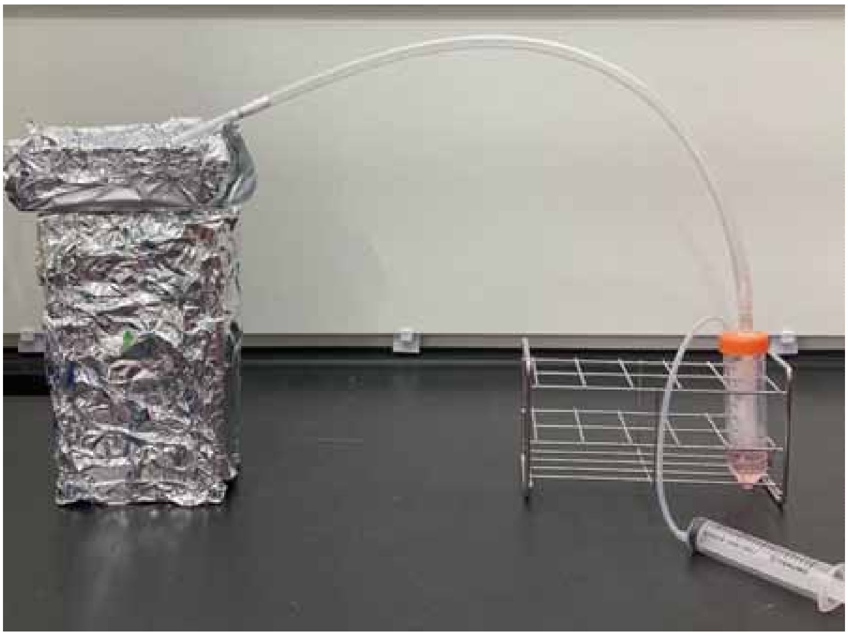

Supplementary Figure 6. Preparation of CSE

Downstream smoke was bubbled through culture medium containing IHGE cells in a disposable tube. Smoke was drawn using an experimenter-operated syringe. Details of the procedure have been presented in a previous study[39].

Supplementary Figure 7. Immunoblotting performed in this study

Figure 1 (Seven Stars)

IB: anti-JAM1 (raw image)

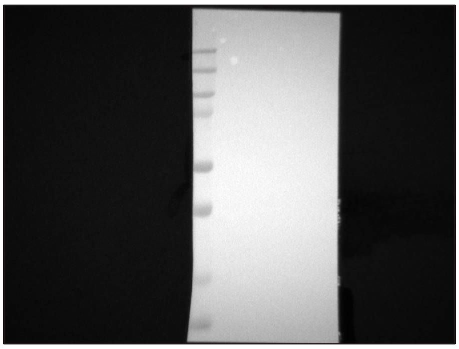

IB: anti-JAM1 (immunoblotting)

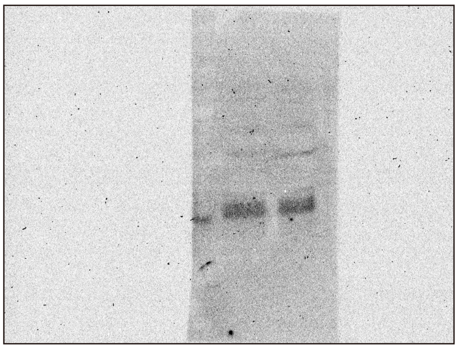

IB: anti-JAM1 (merged and rotated)

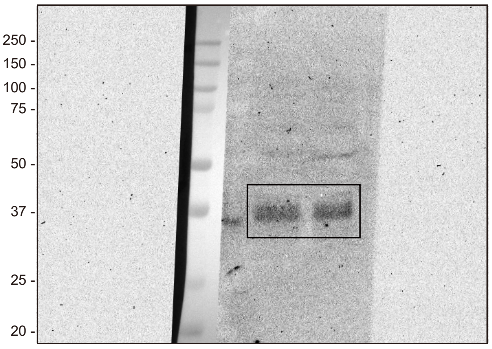

IB: anti-CXADR (raw image)

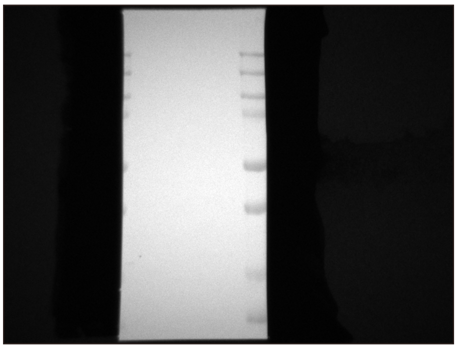

IB: anti-CXADR (immunoblotting)

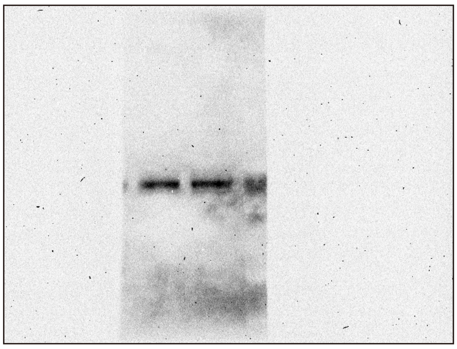

IB: anti-CXADR (merged and rotated)

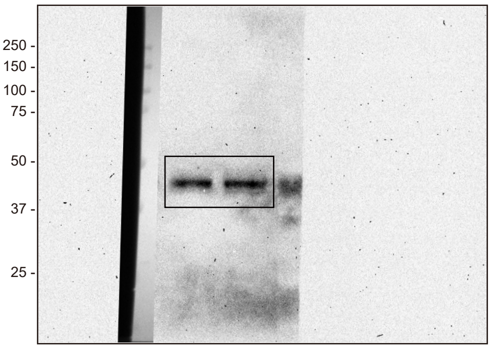

IB: anti-β-ACTIN (raw image)

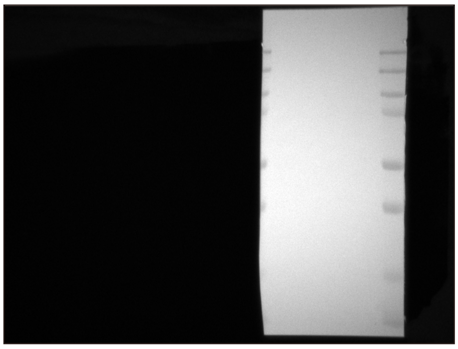

IB: anti-β-ACTIN (immunoblotting)

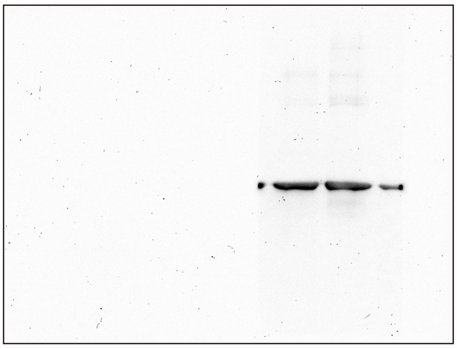

IB: anti-β-ACTIN (merged)

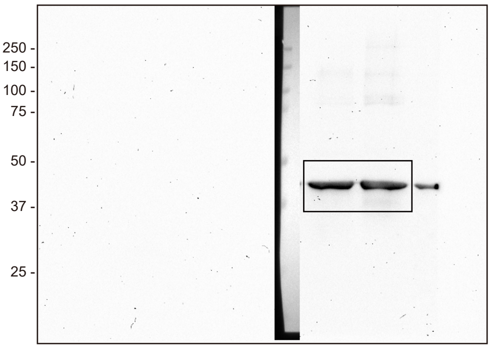

Figure 1 (Kent)

IB: anti-JAM1 (raw image)

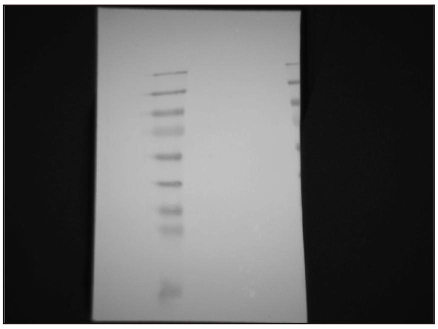

IB: anti-JAM1 (immunoblotting)

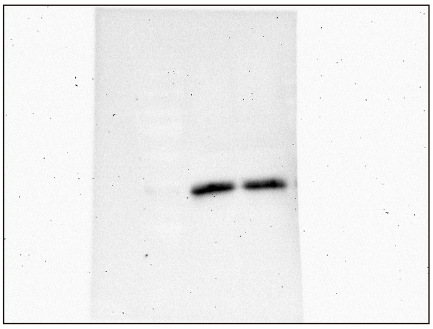

IB: anti-JAM1 (merged and rotated)

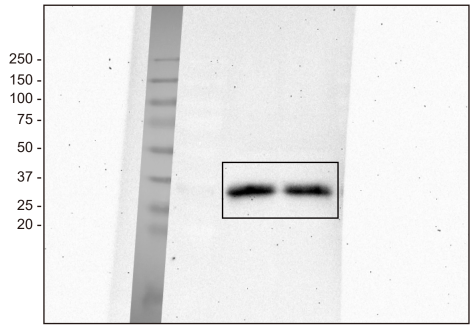

IB: anti-CXADR (raw image)

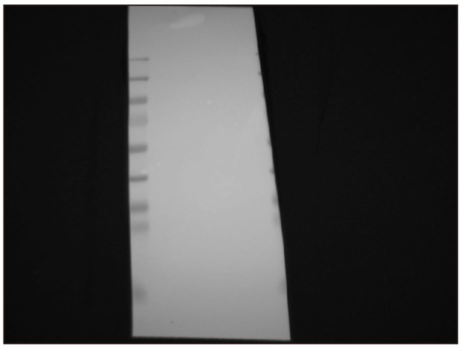

IB: anti-CXADR (immunoblotting)

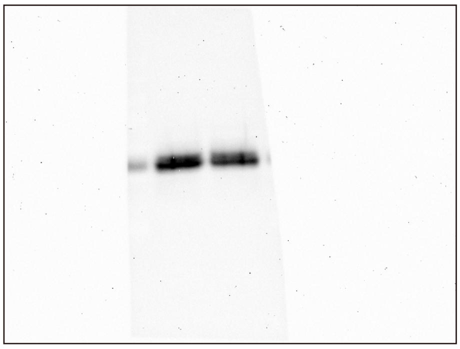

IB: anti-CXADR (merged and rotated)

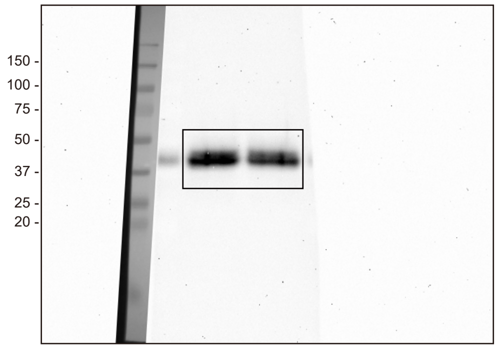

IB: anti-β-ACTIN (raw image)

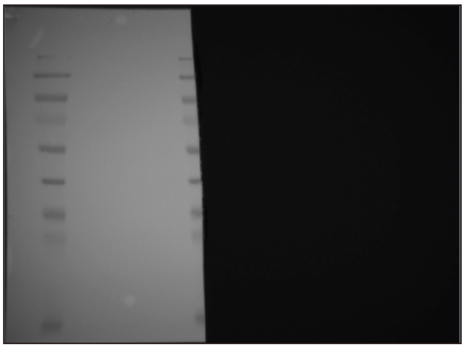

IB: anti-β-ACTIN (immunoblotting)

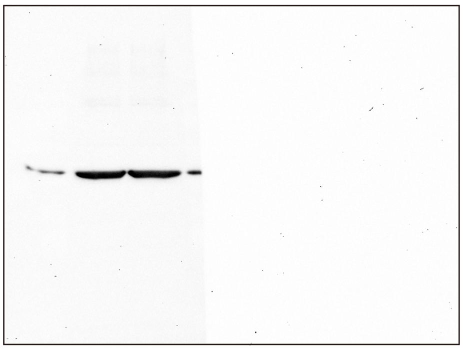

IB: anti-β-ACTIN (merged)

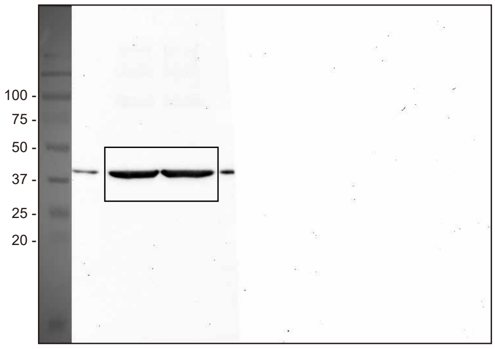

Figure 1 (Marlboro)

IB: anti-JAM1 (raw image)

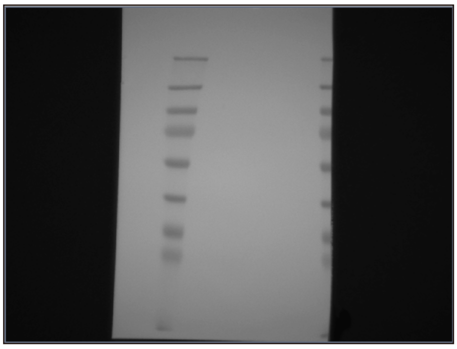

IB: anti-JAM1 (immunoblotting)

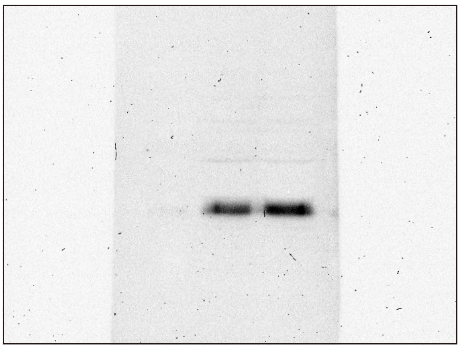

IB: anti-JAM1 (merged)

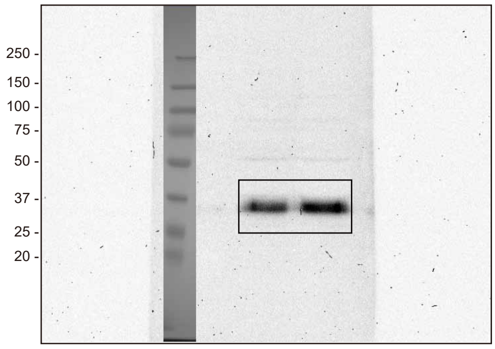

IB: anti-CXADR (raw image)

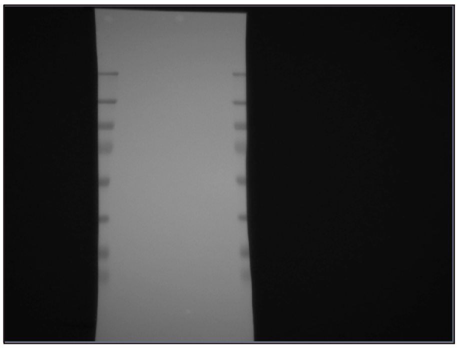

IB: anti-CXADR (immunoblotting)

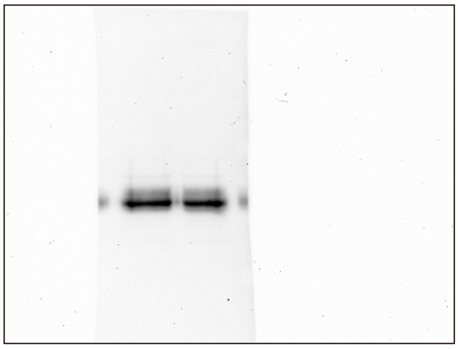

IB: anti-CXADR (merged)

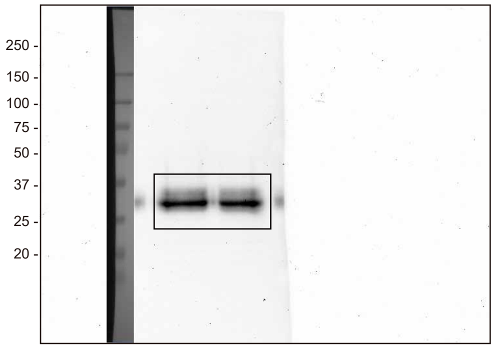

IB: anti-β-ACTIN (raw image)

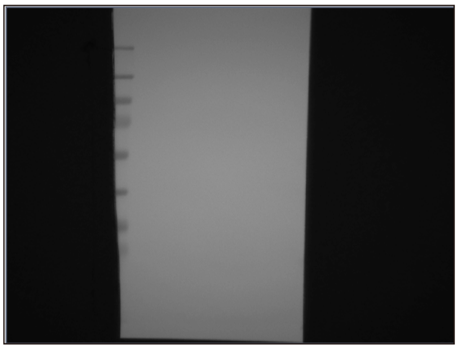

IB: anti-β-ACTIN (immunoblotting)

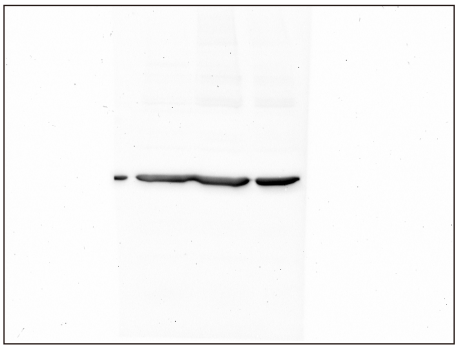

IB: anti-β-ACTIN (merged and rotated)

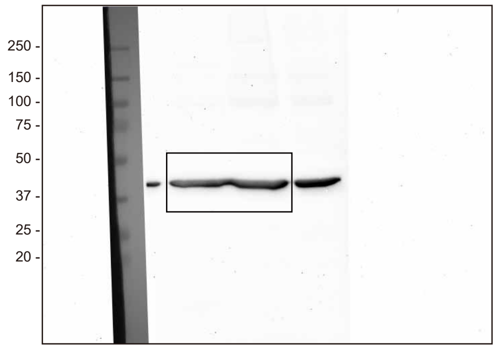

Figure 8

IB: anti-JAM1 (raw image)

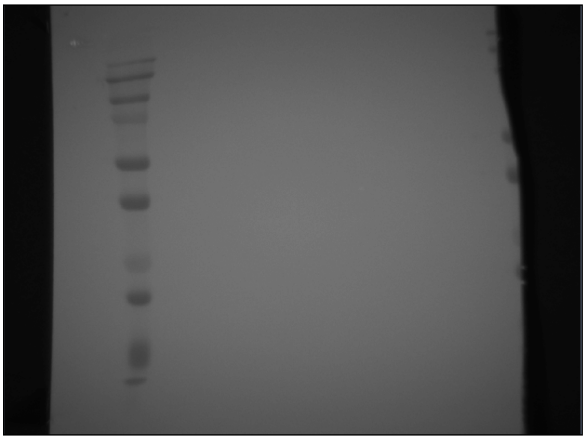

IB: anti-JAM1 (immunoblotting)

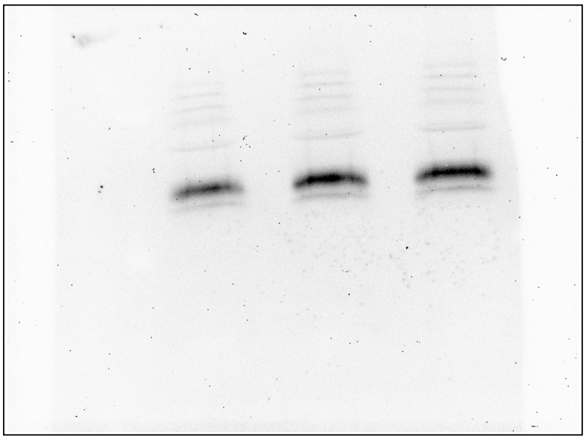

IB: anti-JAM1 (merged and rotated)

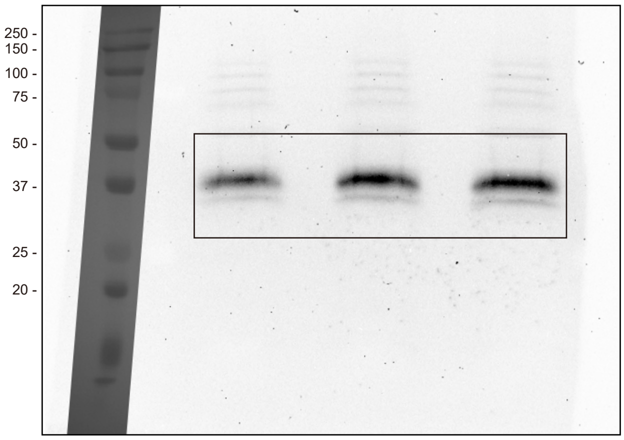

IB: anti-β-ACTIN (raw image)

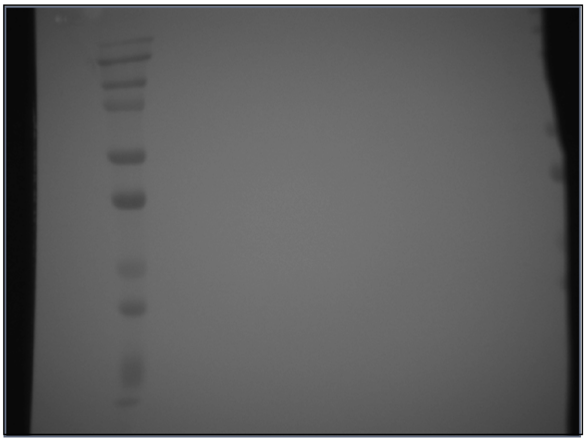

IB: anti-β-ACTIN (immunoblotting)

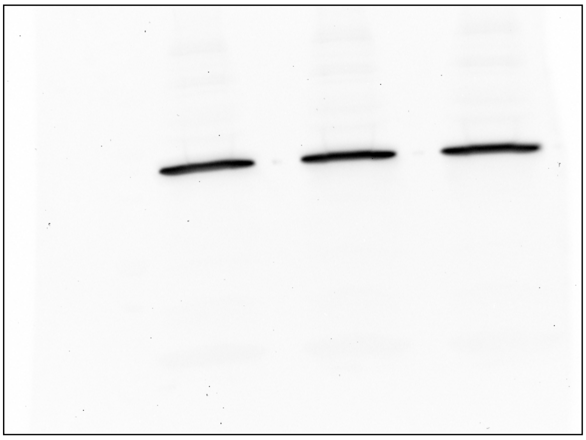

IB: anti-β-ACTIN (merged and rotated)

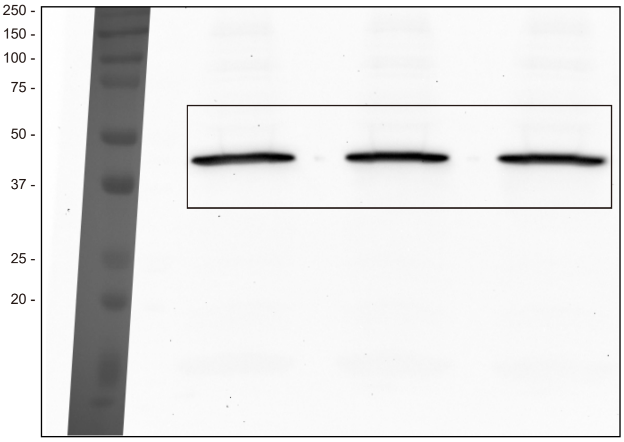

Supplementary Figure 3

IB: anti-JAM1 (raw image)

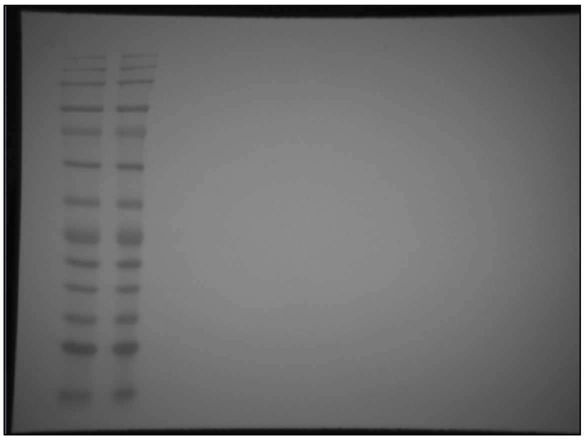

IB: anti-JAM1 (immunoblotting)

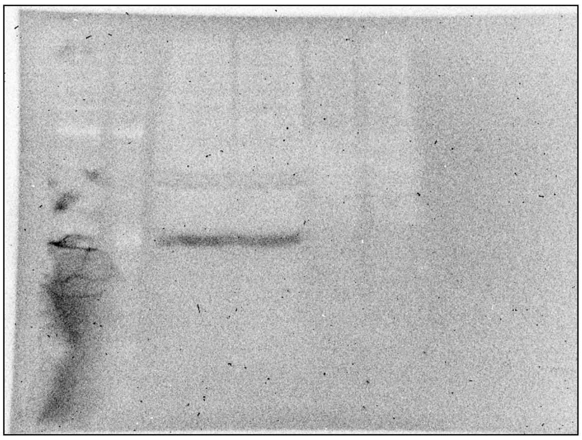

IB: anti-JAM1 (merged)

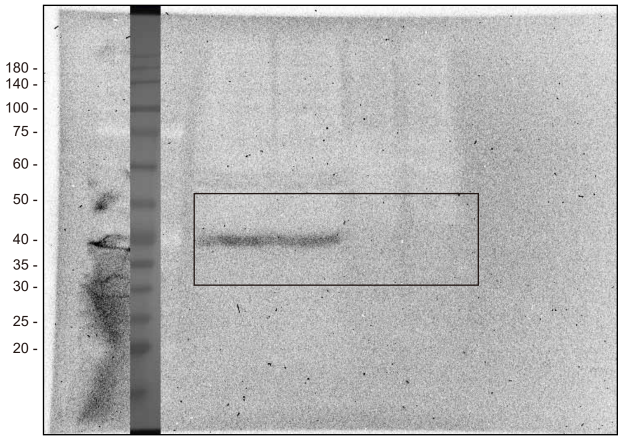

IB: anti-CXADR (raw image)

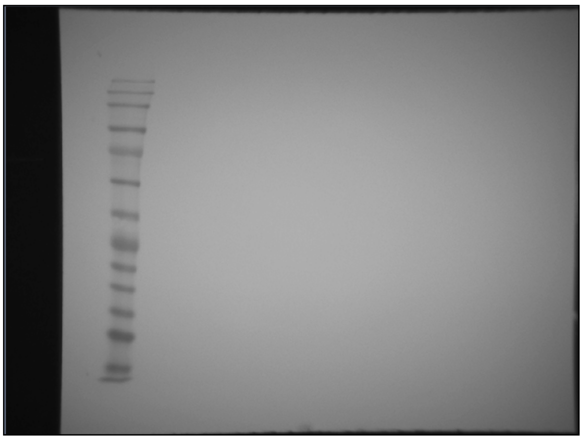

IB: anti-CXADR (immunoblotting)

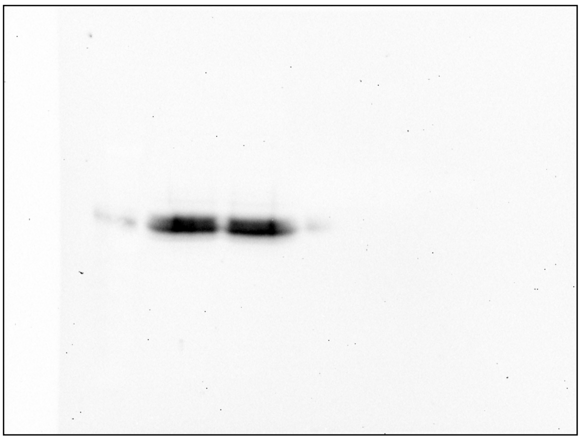

IB: anti-CXADR (merged)

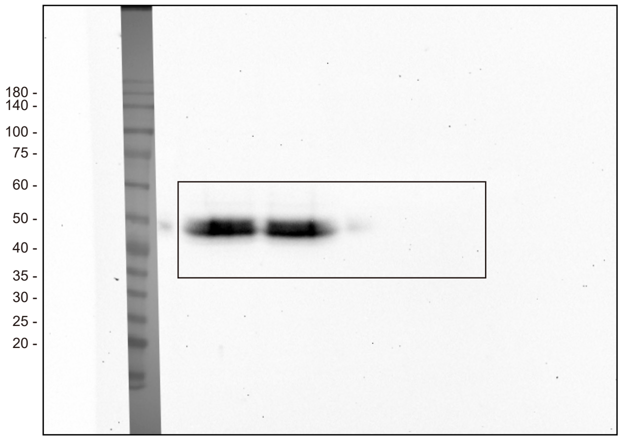

IB: anti-CALNEXIN (raw image)

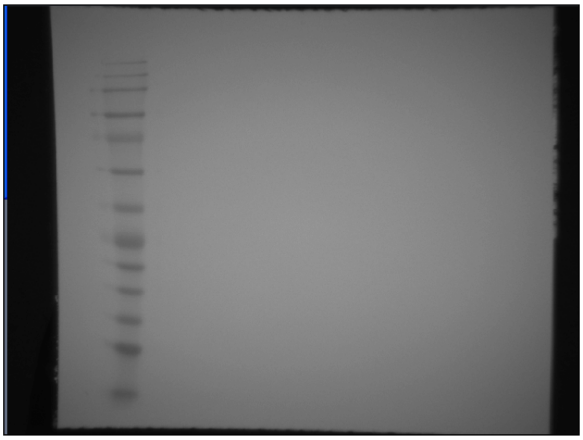

IB: anti-CALNEXIN (immunoblotting)

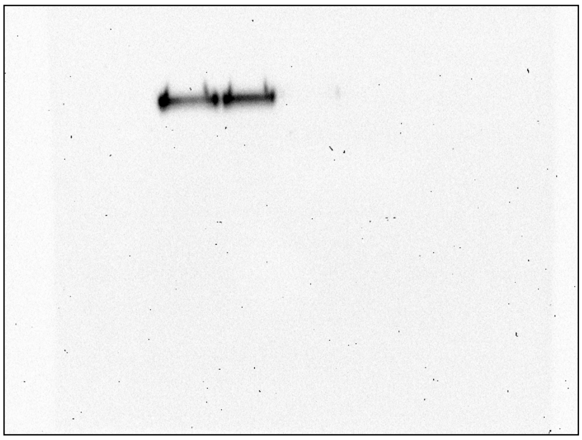

IB: anti-CALNEXIN (merged & rotated)

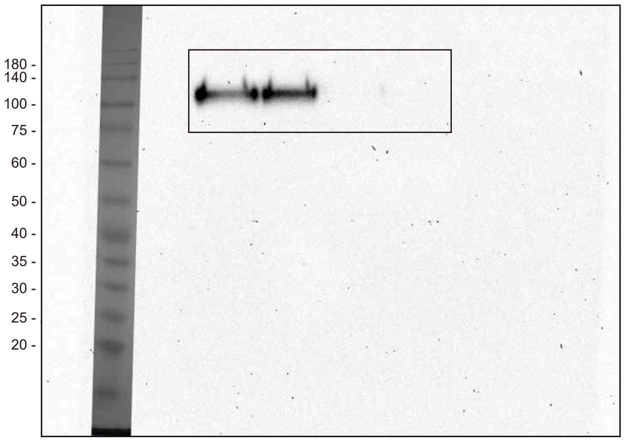

IB: anti-EEA1 (raw image)

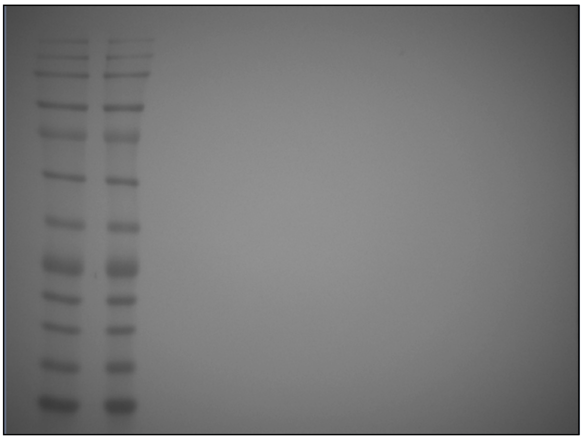

IB: anti-EEA1 (immunoblotting)

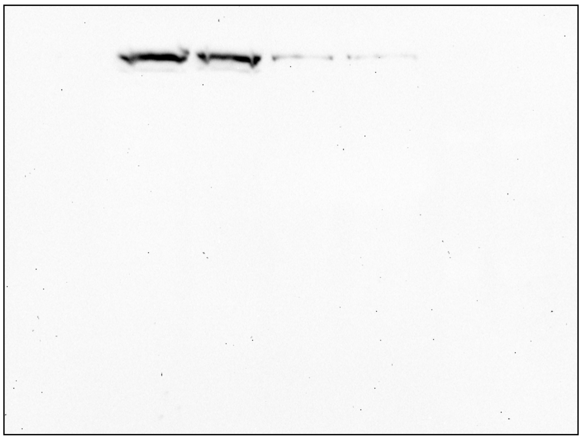

IB: anti-EEA1 (merged)

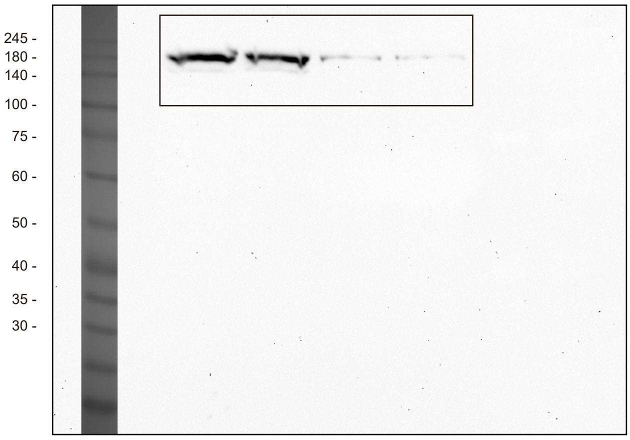

IB: anti-GAPDH (raw image)

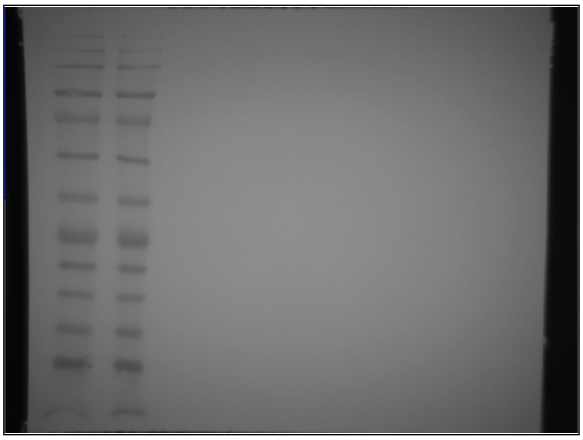

IB: anti-GAPDH (immunoblotting)

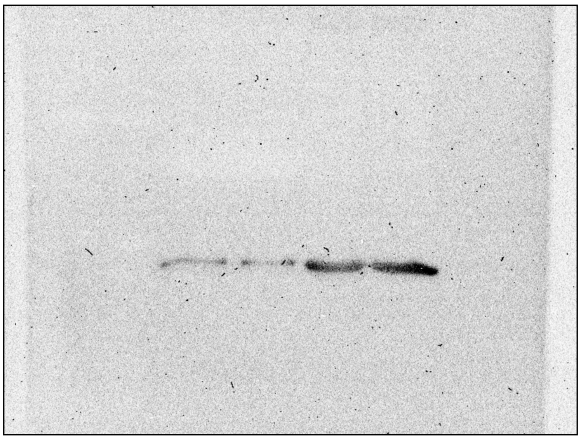

IB: anti-GAPDH (merged & rotated)

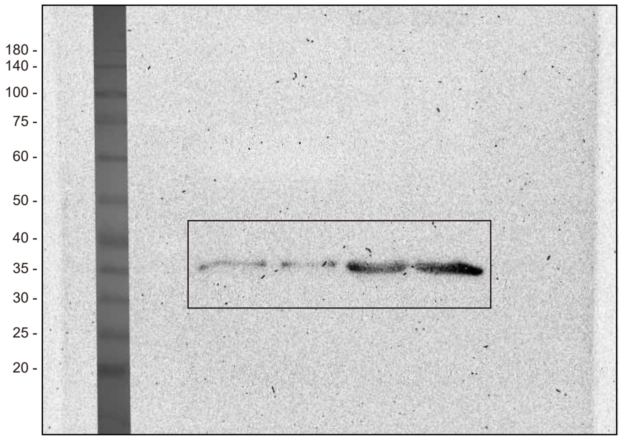

IB: anti-JAM1 (raw image)

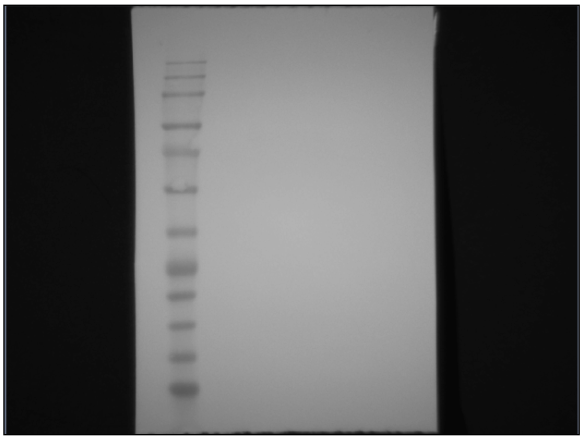

IB: anti-JAM1 (immunoblotting)

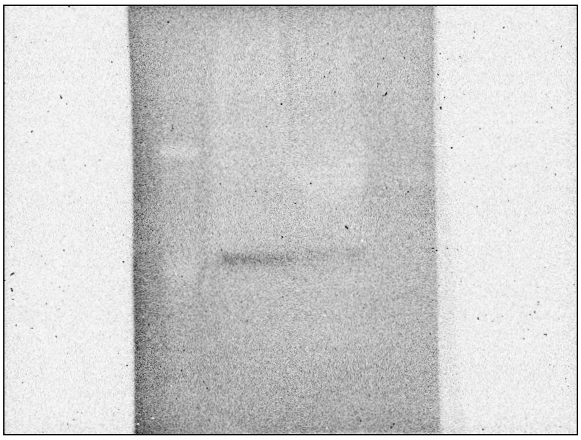

IB: anti-JAM1 (merged and rotated)

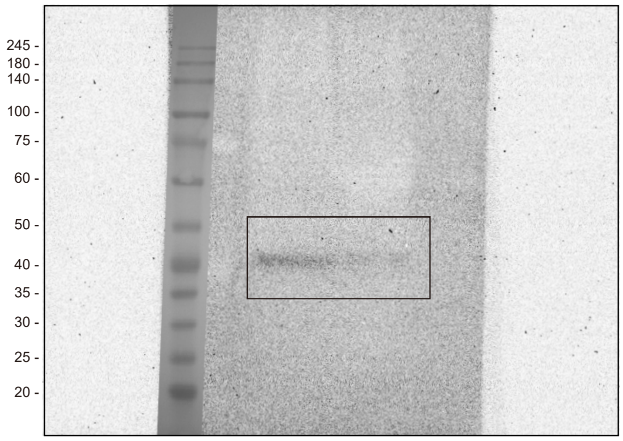

IB: anti-CXADR (raw image)

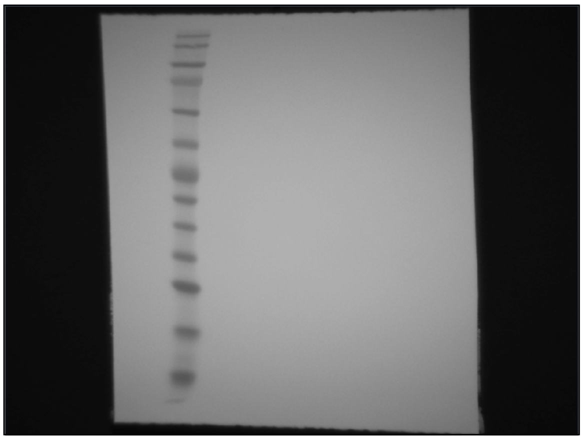

IB: anti-CXADR (immunoblotting)

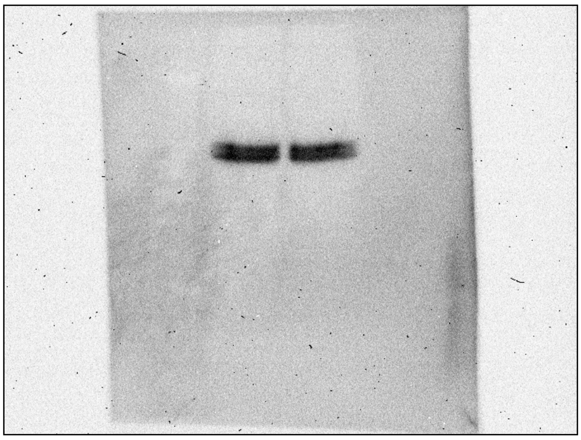

IB: anti-CXADR (merged)

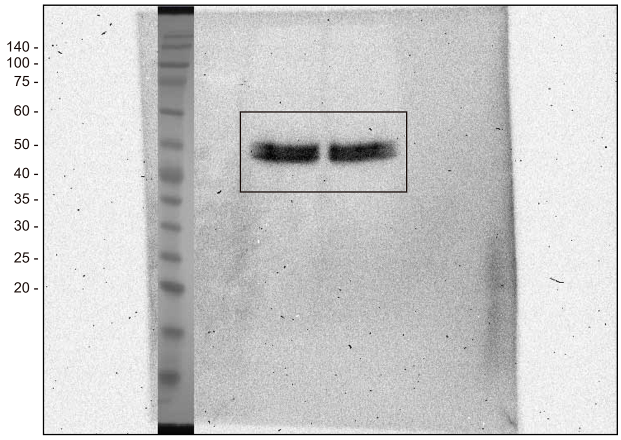

Supplement: Supplementary file 1 — Supplementary Information. [file 41598_2023_36366_MOESM1_ESM.pdf]
